# Multiomics analysis couples mRNA turnover and translational control of glutamine metabolism to the differentiation of the activated CD4^+^ T cell

**DOI:** 10.1038/s41598-022-24132-6

**Published:** 2022-11-16

**Authors:** Louise S. Matheson, Georg Petkau, Beatriz Sáenz-Narciso, Vanessa D’Angeli, Jessica McHugh, Rebecca Newman, Haydn Munford, James West, Krishnendu Chakraborty, Jennie Roberts, Sebastian Łukasiak, Manuel D. Díaz-Muñoz, Sarah E. Bell, Sarah Dimeloe, Martin Turner

**Affiliations:** 1 Immunology Programme, The Babraham Institute, Babraham Research Campus, Cambridge, CB22 3AT UK; 2 Institute of Immunology and Immunotherapy, College of Medical and Dental Sciences, IBR, University of Birmingham, Edgbaston, Birmingham, B15 2TT UK; 3 Cambridge Institute of Therapeutic Immunology and Infectious Disease, Jeffrey Cheah Biomedical Centre, University of Cambridge, Cambridge, CB2 0AW UK; 4 Institute of Metabolism and Systems Research, University of Birmingham, Birmingham, UK; 5 Present Address: IONTAS, The Works, Unity Campus, Cambridge, CB22 3EF UK; 6 Present Address: Nature Reviews Rheumatology, The Campus, 4 Crinan Street, London, N1 9XW UK; 7 Present Address: Immunology Research Unit, GlaxoSmithKline, Gunnels Wood Road, Stevenage, SG1 2NY Herts UK; 8 Present Address: Bioanalysis, Immunogenicity and Biomarkers (BIB), IVIVT, GSK, Stevenage, SG1 2NY UK; 9 Present Address: Discovery Biology, Discovery Science, R&D, AstraZeneca, Cambridge, UK; 10 Present Address: Toulouse Institute for Infectious and Inflammatory Diseases (Infinity), Inserm UMR1291, CNRS UMR5051, University Paul Sabatier, CHU Purpan, BP3028, 31024 Toulouse, France

**Keywords:** CD4-positive T cells, Gene regulation in immune cells, Transcriptomics, RNA-binding proteins, Metabolomics

## Abstract

The ZFP36 family of RNA-binding proteins acts post-transcriptionally to repress translation and promote RNA decay. Studies of genes and pathways regulated by the ZFP36 family in CD4^+^ T cells have focussed largely on cytokines, but their impact on metabolic reprogramming and differentiation is unclear. Using CD4^+^ T cells lacking *Zfp36* and *Zfp36l1*, we combined the quantification of mRNA transcription, stability, abundance and translation with crosslinking immunoprecipitation and metabolic profiling to determine how they regulate T cell metabolism and differentiation. Our results suggest that ZFP36 and ZFP36L1 act directly to limit the expression of genes driving anabolic processes by two distinct routes: by targeting transcription factors and by targeting transcripts encoding rate-limiting enzymes. These enzymes span numerous metabolic pathways including glycolysis, one-carbon metabolism and glutaminolysis. Direct binding and repression of transcripts encoding glutamine transporter SLC38A2 correlated with increased cellular glutamine content in ZFP36/ZFP36L1-deficient T cells. Increased conversion of glutamine to α-ketoglutarate in these cells was consistent with direct binding of ZFP36/ZFP36L1 to *Gls* (encoding glutaminase) and *Glud1* (encoding glutamate dehydrogenase). We propose that ZFP36 and ZFP36L1 as well as glutamine and α-ketoglutarate are limiting factors for the acquisition of the cytotoxic CD4^+^ T cell fate. Our data implicate ZFP36 and ZFP36L1 in limiting glutamine anaplerosis and differentiation of activated CD4^+^ T cells, likely mediated by direct binding to transcripts of critical genes that drive these processes.

## Introduction

Antigen-mediated activation of CD4^+^ T cells triggers profound changes in the transcriptome required for proliferation and differentiation^1^. These are accompanied by rapid increases in aerobic glycolysis, one-carbon metabolism, amino acid uptake and glutaminolysis^2–6^. These metabolic changes meet the increased energetic and biosynthetic demands of proliferation and direct the differentiation and effector functions of T cells^7–14^.

The expression of protein coding genes is regulated by processes that determine the abundance of mRNA, its rate of translation and protein stability^15,16^. Coordination between these processes is critical to ensure the correct timing, peak and duration of gene expression changes following immune challenge. Several studies implicate mRNA stability and direct regulation of translation as playing substantial roles in shaping gene expression upon T cell activation^17–21^. The interplay between transcriptional and post-transcriptional mechanisms is exemplified by T cell metabolic reprogramming: whilst transcription factors such as MYC and HIF1α are implicated in the transcriptional regulation of metabolism during activation^22–24^, translational control is also critically important, as are the presence of pre-existing mRNA and protein reservoirs in naïve T cells^25,26^. Moreover, altered metabolism drives further gene expression changes through both epigenetic and post-transcriptional mechanisms^7,11,27^. In activated CD4^+^ T cells the mechanisms that regulate the interconnection of transcriptional and post-transcriptional processes have yet to be defined, but likely involve sequence specific interactions between mRNAs, microRNAs and RNA binding proteins (RBPs)^28–32^.

The ZFP36 family of RBPs are conserved amongst mammals^33^ and contain tandem CCCH zinc fingers. These bind AU-rich elements (AREs) within the 3’ untranslated region (3’UTR) of mRNA, with the two UAUU motifs within the high affinity UUAUUUAUU ARE^34^ each bound by one of the zinc fingers in ZFP36L2^35^. ZFP36 family proteins promote recruitment of mRNA decay enzymes, including the CCR4-NOT deadenylase complex^36–38^, resulting in destabilisation of their target mRNAs. They have also been implicated in regulating the tempo of translation^39–41^ and subcellular compartmentalisation of proteins^42^.

ZFP36L1 and ZFP36L2 play essential roles in T cell development^43,44^. In memory T cells, ZFP36L2 has been implicated in repressing the translation of cytokine genes, including *Ifng*^45^. In mature T cells, expression of ZFP36 and ZFP36L1 is induced following T cell activation^46,47^. Deletion of *Zfp36* in all cells of the mouse resulted in increased mRNA and protein expression for both interleukin 2 (IL-2) and interferon gamma (IFNγ), accompanied by increased mRNA stability in mixed populations of splenic cells^48,49^. These studies also demonstrated binding of ZFP36 to AREs within the 3’UTRs of each gene. Whilst one study suggested that elevated IFNγ production in *Zfp36*-deficient CD8^+^ T cells was a consequence of an increased proportion of effector T cells and not an increased ability of T cells to produce IFNγ^50^, the most recent and comprehensive genome-wide study of ZFP36 in T cells indicated that numerous cytokines, including *Ifng*, are direct ZFP36 targets and confirmed a modest but significant increase in IFNγ protein in T cells upon germline deletion of *Zfp36*^46^. Their data suggested that ZFP36 negatively regulates both mRNA abundance and translation of its target genes, and additionally identified genes involved in activation, proliferation and effector function as direct ZFP36 targets^46^. Although these studies show roles for *Zfp36* in T cell activation, the contribution of the other paralogs is mostly unknown, and some roles of the ZFP36 family may have been masked by redundancy between ZFP36 and ZFP36L1^51^. Roles for the ZFP36 family in the metabolism of immune cells have not yet been identified.

Here, we employed a multiomics approach to investigate the function of ZFP36 family members in the activation of undifferentiated naïve mouse CD4^+^ T cells. We measured the global impact of deletion of *Zfp36* and *Zfp36l1* on transcription, transcript stability, mRNA abundance and translation. By distinguishing direct from indirect target genes, we implicate ZFP36 family RBPs in limiting glutamine metabolism and differentiation of naïve CD4^+^ T cells towards Th1 and cytotoxic phenotypes. We propose that this is mediated in part by the direct repression of critical genes in metabolic pathways.

## Results

### Zfp36 and Zfp36l1 regulate transcription, mRNA stability and translation

Given the rapid induction of ZFP36 and ZFP36L1^46^, we activated naïve CD4^+^ T cells isolated from *Zfp36*^fl/fl^/*Zfp36l1*^fl/fl^*Cd4*^cre^ (dKO) and matched *Zfp36*^fl/fl^/*Zfp36l1*^fl/fl^ (control) mice for 24 h with anti-CD3 and anti-CD28 antibodies. This timepoint is one at which T cells are growing in preparation for the onset of cell division and have undergone substantial metabolic reprogramming^2^. At this time, ZFP36/ZFP36L1 are still expressed^46^, and we anticipated that they would regulate, by direct or indirect means, the delayed primary and secondary response genes that coordinate proliferation and differentiation^52^. Following activation, dKO and control CD4^+^ T cells expressed CD69 similarly, indicating that differences in gene expression we detected were not driven by differences in the proportion of activated cells (Supplementary Fig. S1a). The efficient deletion of both *Zfp36* and *Zfp36l1* was confirmed by qRT-PCR and *Zfp36l2* mRNA abundance was unchanged (Supplementary Fig. S1b). To establish whether the redundancy between ZFP36 and ZFP36L1 observed in CD8^+^ T cells^51^ was also apparent in CD4^+^ T cells, we first performed RNA-seq to measure mRNA abundance in the dKO compared with control, and analysed this data together with RNA-seq from single KO models (*Zfp36*^fl/fl^*Cd4*^cre^ and *Zfp36l1*^fl/fl^*Cd2*^cre^, each compared with corresponding floxed controls). Whilst the *Zfp36l1* single KO utilises CD2- rather than CD4-Cre, we expect this to have minimal impact on our results, since in both cases deletion occurs during thymic development, and should be complete within the isolated population of naïve CD4^+^ T cells. Differential expression analysis revealed substantial changes in the expression of many genes in the dKO (FDR-adjusted *p* value < 0.05), whilst the single KO models showed far fewer changes, of smaller magnitude (Supplementary Fig. S1c–e; Supplementary Table S1). For more detailed analysis of post-transcriptional mechanisms, we therefore chose to focus solely on the dKO.

For RNA synthesis measurements, we added 4-thiouridine for the final hour of the activation period, after which we separated the RNA into labelled (newly-synthesised), unlabelled (pre-existing) and total fractions to enable us to additionally infer the stability of transcripts based on the ratio between the newly-synthesised and total RNA^53^. We also determined ribosome association (a surrogate for translation) in addition to mRNA abundance.

**Figure 1:**
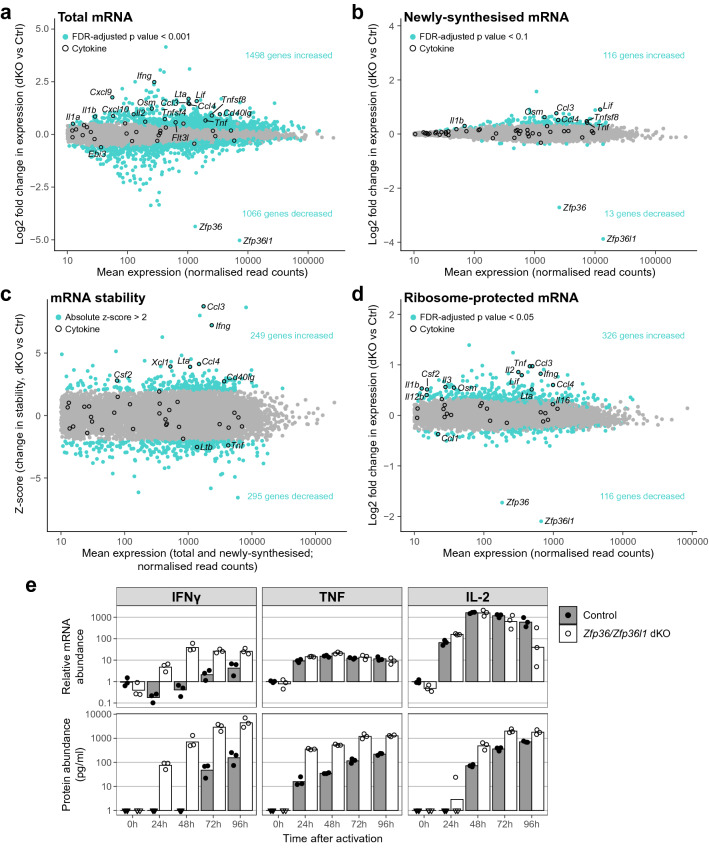
*Zfp36* and *Zfp36l1* regulate transcription, mRNA stability and translation. (**a, b, d**) MA plots showing changes in total (**a**), newly-synthesised (**b**) and ribosome-protected mRNA (**d**) in dKO activated CD4^+^ T cells compared with floxed controls. (**c**) Z-scores for change in mRNA stability in dKO activated CD4^+^ T cells compared with floxed controls. Z-scores and p values were calculated based on the standard deviation of genes with similar mean expression. (**e**) Measurements of RNA and protein abundance for IFNγ, TNF and IL-2 in control and dKO activated CD4^+^ T cells. RNA abundance was measured by quantitative RT-PCR, whilst protein accumulation in culture supernatants was measured using a BD cytometric bead array. Inverted triangular points indicate that abundance was below the limit of detection.

Analysis of mRNA abundance using a stringent threshold (FDR-adjusted *p* value < 0.001) identified 1498 genes with increased and 1066 genes with decreased abundance in dKO CD4^+^ T cells compared with controls (Fig. 1a; Supplementary Table S2). Fewer genes were significantly altered in transcription (Fig. 1b; Supplementary Table S3), stability (Fig. 1c; Supplementary Table S4) or translation (Fig. 1d; Supplementary Table S5). This is true even when lower stringency thresholds are applied, likely reflecting the lower sensitivity of RNA labelling and ribosome profiling methods. Examination of cytokine-encoding mRNAs showed increased abundance of *Ccl3*, *Ccl4* and *Ifng* mRNA (Fig. 1a), each of which is bound and repressed by ZFP36^49,54,55^. This is matched by increased transcript stability and translation (Fig. 1c, d), suggesting that in dKO cells, their increased abundance is partly due to more stable mRNA. Interestingly, for *Ccl3* and *Ccl4*, we observed a contribution of increased transcription (Fig. 1b); for *Ifng* transcripts, the highly variable measurements of newly-synthesised mRNA preclude conclusions about its transcription. In contrast, increases in *Tnf* and *Il2* ribosome-protected mRNA outweigh the modest increases in their transcription and/or mRNA abundance, and neither exhibited increased mRNA stability (Fig. 1a–d). These results suggest that in CD4^+^ T cells, at the timepoint we chose, the repression of translation is the principal mechanism by which the ZFP36 family represses TNF and IL-2. This is consistent with findings in *Zfp36* single KO CD4^+^ T cells 4 h after activation^46^, in which TNF was repressed at the level of translation but not mRNA stability. The different mechanisms regulating *Ifng*, *Tnf* and *Il2* in dKO CD4^+^ T cells were supported by qRT-PCR and protein measurements over an extended time course (Fig. 1e). Thus, ZFP36/ZFP36L1 repress the expression of cytokines and chemokines by limiting their transcription, mRNA stability and translation.

### Genes derepressed following loss of ZFP36/ZFP36L1 cluster into five modes of regulation

**Figure 2:**
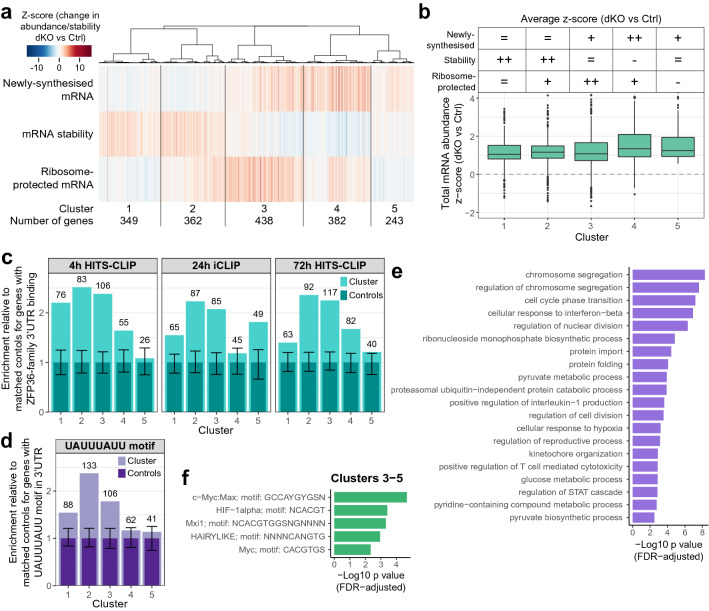
Loss of *Zfp36* and *Zfp36l1* results in distinct modes of gene regulation transcriptome-wide. (**a**) Heatmap showing z-scores for genes with an increase in their total, newly-synthesised or ribosome-protected mRNA, or mRNA stability. Genes were hierarchically clustered based on the correlation between z-scores for the latter three of these measures. (**b**) Top: summary of the clusters shown in **a** (+ median z-score > 0.4; - median z-score < -0.4; ++ median z-score > 1.2); Bottom: boxplot showing the changes in mRNA abundance for dKO compared with control CD4^+^ T cells for genes in each cluster. (**c, d**) Enrichment for genes identified as ZFP36/ZFP36L1 targets in each CLIP dataset (**c**) or with a UAUUUAUU motif in their 3’UTR (**d**) within each cluster in **a** relative to expression- and 3’UTR length-matched controls. Light turquoise/purple bars represent the number of target genes/genes with a motif in the cluster, normalised to the median across 100 sets of controls (dark turquoise/purple bars). Error bars represent normalised 5% and 95% quantiles for the control sets; numbers above each bar indicate the absolute number of target genes/genes containing a motif in the cluster. (**e**) Gene ontology (biological process) analysis of all increased genes depicted in **a**. Significant GO terms with enrichment > 2 were identified using GOrilla and reduced using REVIGO. The top 20 terms (ranked by -log10 FDR-adjusted p value) are shown. (**f**) Enriched binding motifs and associated transcription factors from gProfiler analysis of genes comprising clusters 3–5 against the TRANSFAC database.

To understand the extent and nature of this multimodal repression of gene expression we focussed on genes with increased transcription, stability or translation. This will include the majority of direct ZFP36-family targets which are no longer repressed in dKO cells, but also genes increased by indirect mechanisms. We grouped these mRNAs according to their modes of regulation and selected those that displayed an increase in newly-synthesised mRNA (FDR-adjusted *p* value < 0.1; 116 genes); total mRNA (FDR-adjusted *p* value < 0.001; 1498 genes); mRNA stability (z-score > 2; 249 genes); or ribosome-protected mRNA (FDR-adjusted *p* value < 0.05; 326 genes) for further consideration. The significance thresholds were chosen to ensure contribution to the analysis of genes altered in each dataset. Hierarchical clustering based on the correlations between z-scores for transcription, stability and translation assigned genes to five clusters with distinct regulatory modes (Fig. 2a; summarised in 2b; Supplementary Table S6). Clusters 1 and 2 contain genes with increased stability. Cluster 2 genes additionally show increased translation, and this group includes the cytokines *Ifng, Csf2, Lta* and *Cd40lg*. Cluster 3 contains genes for which increased translation typically outweighs increases in the other measures. This cluster includes *Tnf*, *Il2,* and the cytotoxicity genes *Gzmb* and *Prf1*, as well as several transcription factors important in T cell activation and differentiation, such as *Tbx21*, *Eomes*, *Notch1*, *Gata3*, *Irf8, Myc* and *Hif1a* (Supplementary Table S6). Clusters 3–5 include genes with increased transcription. We noted that genes with a large increase in transcription frequently displayed decreased mRNA stability. This effect might arise from the action of a homeostatic mechanism destabilising transcripts with an increased transcription rate as found in yeast^56^. However, it could also be evident for mRNAs whose abundance was far from steady state during the 1 h 4-thiouridine labelling period, because the calculation of mRNA stability, based on the ratio of newly-synthesised to total mRNA, assumes that both are at steady state. Whilst we expect many mRNAs to be close to steady state by 24 h following activation, for genes that are not and that additionally display a substantial increase in transcription in the dKO, this could result in mRNA abundance being further from steady state in the dKO than in the control, leading to a greater underestimation of mRNA stability and thus an apparent decrease in stability. All five clusters displayed a similar distribution in their increase in total mRNA (Fig. 2b), indicating that increased transcription and mRNA stability both drive changes in mRNA abundance in the dKO cells. Although this approach does not distinguish the direct from indirect effects of ZFP36/ZFP36L1, it reveals the extensive repression of transcription by the ZFP36 family and that the repressive effects of ZFP36 family members on translation are frequently uncoupled from changes in mRNA abundance.

### ZFP36 and ZFP36L1 act directly to reduce transcript stability and/or ribosome-association

To distinguish direct from indirect effects of ZFP36-family proteins on derepressed genes we identified transcripts bound by ZFP36L1 in CD4^+^ T cells activated for 24 h with anti-CD3 and anti-CD28 by Individual-nucleotide resolution UV CrossLinking and ImmunoPrecipitation (iCLIP); hereafter 24 h iCLIP (Supplementary Table S7). To complement this and improve the sensitivity and robustness of target detection, we also reanalysed published HITS-CLIP data of transcripts detected with a pan-ZFP36-family antibody from CD4^+^ T cells that had been activated by anti-CD3 and antigen-presenting cells for 4 h (hereafter 4 h HITS-CLIP) or for 72 h with PMA + ionomycin added for the final 2 h (hereafter 72 h HITS-CLIP)^46^. We obtained separate lists of 3’UTR targets for each, with at least 50% of genes identified from a given dataset also found in at least one other (Supplementary Table S7). Clusters 2 and 3, which show the strongest increases in stability and translation, respectively, were consistently enriched for transcripts with 3’UTRs bound by ZFP36/ZFP36L1, with over two-fold more targets relative to control gene sets matched for mRNA abundance and 3’UTR length (Fig. 2c). The enrichment was lower in cluster 1, in which increased stability was not typically accompanied by increased translation. For clusters 4 and 5, there was enrichment in only some of the CLIP datasets, indicating that transcriptional regulation is frequently an indirect effect of ZFP36/ZFP36L1. Enrichment for the ARE UAUUUAUU, which comprises a high affinity ZFP36-family binding motif^34^, was also greatest for cluster 2 (Fig. 2d). The consistent enrichment for 3’UTR binding and sequence motifs identified the distinguishing features of high confidence direct targets: these show increased mRNA stability and translation when ZFP36 and ZFP36L1 are absent.

### ZFP36 family proteins directly target genes encoding key metabolic enzymes

Gene ontology analysis of all genes that were derepressed in the dKO mice revealed numerous pathways, including chromosome segregation and the cell cycle. Several metabolic pathways were also highlighted, with strong enrichment for terms related to biosynthesis of pyruvate or nucleotides (Fig. 2e) and modest enrichment for serine, glutamine and tetrahydrofolate biosynthesis (Supplementary Table S8). ZFP36 has been implicated in the post-transcriptional regulation of several transcripts important in metabolism, including iron uptake, mitochondrial iron/sulfur-containing proteins and glycolysis^57–60^. Some of the iron-related transcripts displayed 3’UTR binding of ZFP36-family RBPs in CD4^+^ T cells and increased mRNA abundance in the dKO. These included *Tfrc* encoding the transferrin receptor, which also displayed increased stability but not translation, *Uqcrfs1* encoding a component of complex III of the electron transport chain and *Aco2* encoding a tricarboxylic acid (TCA) cycle enzyme (Supplementary Tables S2–S7). In contrast, *Ndufs1* encoding a component of complex I of the electron transport chain and *Lias* encoding an enzyme which synthesises lipoic acid, a mitochondrial antioxidant, were not detected by CLIP, nor was their expression substantially altered in dKO CD4^+^ T cells. Within the glycolytic pathway, both *Hk2*, which encodes the rate-limiting enzyme hexokinase, previously reported to be repressed by ZFP36 in human cancer cells^60^, and *Pfkfb3*, which converts fructose-6 phosphate to fructose-2,6 bisphosphate, an allosteric promoter of glycolysis, were bound at AREs within their 3’UTR (Supplementary Fig. S2a). *Pfkfb3* mRNA contains a destabilising ARE^61^ that is bound by ZFP36 in human cancer cells^59^. Furthermore, amongst the direct 3’UTR targets in CD4^+^ T cells were the transcription factors cMYC and HIF1α (Supplementary Fig. S2a), which are important in the regulation of metabolism during CD4^+^ T cell activation^22–24^ and target many glycolytic genes, including both *Hk2* and *Pfkfb3*^62–65^. This is consistent with RNA pull-down experiments and reporter gene studies in tumour cells^66,67^. Moreover, we found an enrichment for DNA motifs bound by cMYC or HIF1α amongst genes with increased transcription (clusters 3–5; Fig. 2f; Supplementary Table S8), suggesting their increased activity. Taken together, these data suggested that ZFP36/ZFP36L1 may limit anabolic metabolism in CD4^+^ T cells indirectly via transcription factors, reinforced by binding of ZFP36 or ZFP36L1 within the 3’UTR of key targets, and prompted us to further examine the regulation of anabolic pathways by the ZFP36 family.

**Figure 3:**
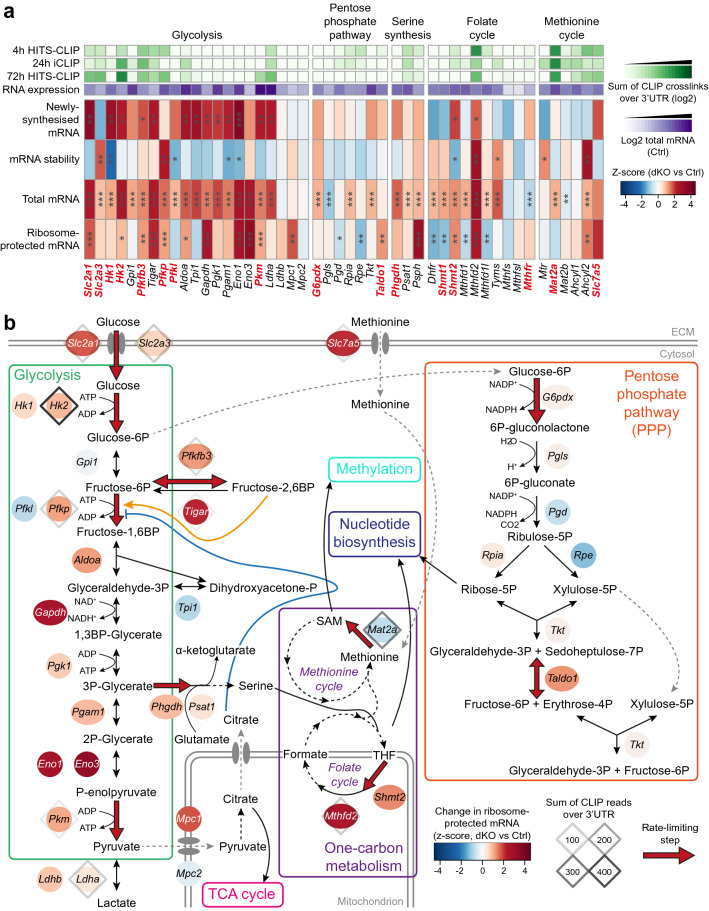
Deletion of *Zfp36* and *Zfp36l1* results in perturbed gene expression for transcripts related to glucose metabolism. (**a**) Heatmap showing z-scores for changes in transcriptomic data for genes in the indicated metabolic pathways. The log2-transformed sum of crosslinks (with FDR < 0.25) over the 3’UTR for each CLIP dataset, normalised to the 90% quantile for detected genes, and mean mRNA abundance for control samples are displayed. Significance from DESeq2 analysis (or as described in methods for stability) is indicated: *** FDR-adjusted p value < 0.05; ** raw p value < 0.05; * raw p value < 0.1. Genes implicated as rate-limiting are indicated in bold, red font. (**b**) Schematic of glucose metabolic pathways, in which the genes encoding enzymes and transporters are depicted as ovals; the colour represents the z-score for ribosome-protected mRNA for that gene. Diamonds indicate genes that were detected in the 72 h HITS-CLIP data; the shade represents the sum of crosslinks (with FDR < 0.25) over the 3’UTR. Red arrows indicate steps implicated as rate-limiting.

Close examination of genes related to glucose uptake and metabolism revealed increased transcription and translation of the majority of these (Fig. 3), accompanied by ZFP36-family binding to conserved AREs within the 3’UTRs of *Slc2a1* and *Slc2a3*, which encode the GLUT1 and GLUT3 glucose transporters (Supplementary Fig. S2b–e). *Slc2a3* mRNA stability was increased in dKO CD4^+^ T cells, but *Slc2a1* mRNA was not; however, a strong increase in its newly-synthesised mRNA might have masked an effect on stability (Fig. 3a). There was also direct binding to a conserved ARE within the 3’UTR of *Pfkp* (Supplementary Fig. S2f,g), in which binding was accompanied by increased transcript stability (Fig. 3a). Both glucose transport and phosphofructokinase have been implicated as rate-limiting for glycolysis^68^.

Serine and methionine contribute to one-carbon metabolism, which plays critical roles in T cell differentiation and function^2,5,27,69^. *Mthfd2* from the folate cycle and *Mat2a* from the methionine cycle displayed clear 3’UTR binding to conserved AREs by ZFP36-family RBPs (Supplementary Fig. S3a–d), and many genes contributing to serine synthesis and one-carbon metabolism showed increased mRNA abundance in dKO CD4^+^ T cells, albeit *Mat2a* mRNA stability and translation was unaltered (Fig. 3a). *Mthfd2* is implicated in cell proliferation and enhanced flux through the mitochondrial branch of the folate cycle^70–72^ and has been identified as an important metabolic checkpoint in CD4^+^ T cells^73^. It showed particularly strong increases in mRNA stability, abundance and translation (Fig. 3).

**Figure 4:**
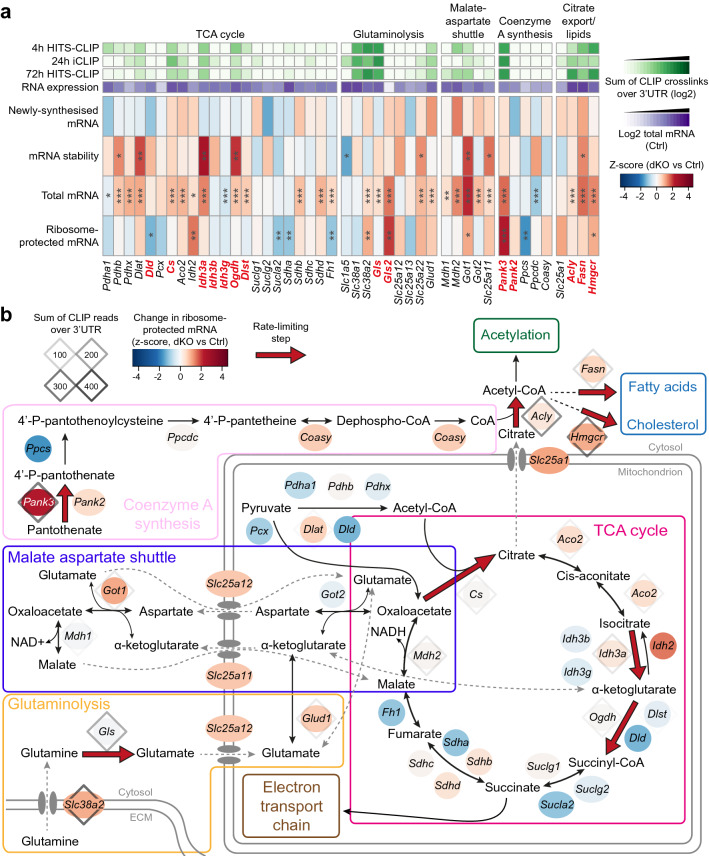
Deletion of *Zfp36* and *Zfp36l1* results in perturbed gene expression for transcripts related to glutamine metabolism and the TCA cycle. (**a**) Heatmap showing z-scores for changes in transcriptomic data for genes in the indicated metabolic pathways. The log2-transformed sum of crosslinks (with FDR < 0.25) over the 3’UTR for each CLIP dataset, normalised to the 90% quantile for detected genes, and mean mRNA abundance for control samples are displayed. Significance from DESeq2 analysis (or as described in methods for stability) is indicated: *** FDR-adjusted p value < 0.05; ** raw p value < 0.05; * raw p value < 0.1. Genes implicated as rate-limiting are indicated in bold, red font. (**b**) Schematic of the TCA cycle and connected metabolic pathways, in which the genes encoding enzymes and transporters are depicted as ovals; the colour represents the z-score for ribosome-protected mRNA for that gene. Diamonds indicate genes that were detected in the 72 h HITS-CLIP data; the shade represents the sum of crosslinks (with FDR < 0.25) over the 3’UTR. Red arrows indicate steps implicated as rate-limiting.

**Figure 5:**
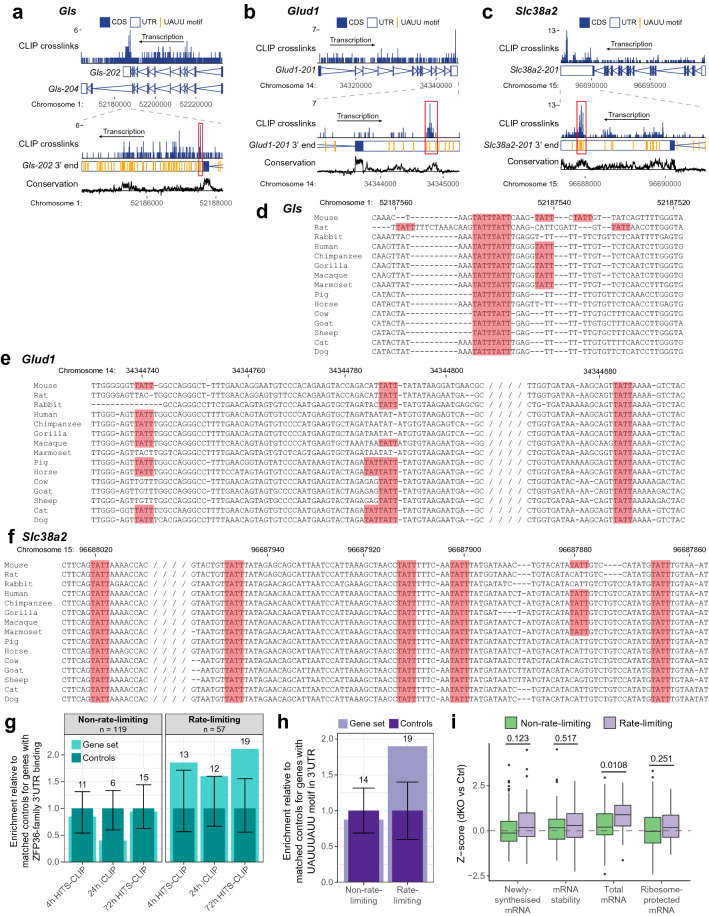
ZFP36 family proteins directly target genes encoding rate-limiting metabolic enzymes. (**a**-**c**) ZFP36/ZFP36L1 crosslinks over the indicated *Gls* (**a**), *Glud1* (**b**) and *Slc38a2* (**c**) transcripts in the 72 h HITS-CLIP data. The top panel depicts the whole transcript(s); the bottom panel is zoomed in on the 3’UTR and shows occurrences of the UAUU motif as vertical orange lines. Conservation tracks represent phyloP scores from a 60-way multiple alignment averaged over a sliding 7 bp window. (**d-f**) Multiple sequence alignments of the regions indicated by red boxes in **a-c**, over which ZFP36/ZFP36L1 binding was detected. TATT (UAUU) motifs are highlighted. Genomic coordinates for the mouse 3’UTR are indicated. (**g, h**) Enrichment for genes identified as ZFP36/ZFP36L1 targets in each CLIP dataset (**g**) or with a UAUUUAUU motif in their 3’UTR (**h**), amongst genes encoding rate-limiting or non-rate-limiting metabolic enzymes and transporters, relative to expression- and 3’UTR length-matched controls. Light turquoise/purple bars represent the number of target genes/genes with a motif in the set, normalised to the median number across 100 sets of controls (dark turquoise/purple bars). Error bars represent normalised 5% and 95% quantiles for the control sets; numbers above each bar indicate the absolute number of target genes/genes containing a motif in the set. (**i**) Z-scores for the change in mRNA abundance or stability of rate-limiting and non-rate-limiting genes upon dKO of *Zfp36* and *Zfp36l1*. P values were calculated using an unpaired Student’s t-test and FDR-correction.

We also found increased expression of genes involved in glutamine metabolism. *Slc38a2*, which encodes the SNAT2 glutamine transporter; *Gls*, a rate-limiting enzyme of glutaminolysis; and *Glud1*, a mitochondrial enzyme that converts glutamate to α-ketoglutarate, all showed increased mRNA abundance (Fig. 4) and were bound by ZFP36-family RBPs to conserved UAUU motifs in their 3’UTR (Fig. 5a–f). *Slc38a2* translation was also increased, and we observed positive z-scores for stability of *Gls* transcripts and translation of *Glud1*, as well as transcription of *Slc38a2* and *Glud1*, albeit these did not reach statistical significance. The lack of statistically significant changes might reflect the lower sensitivity of the 4-thiouridine labelling and ribosome profiling methods. It is also possible that altered transcription, stability or translation might occur at an earlier timepoint during activation, perhaps when ZFP36-family protein expression is at its peak. Thus, these data suggest that the ZFP36-family might regulate glutamine uptake and utilisation in CD4^+^ T cells, with direct targeting of key transcripts by the ZFP36 family potentially contributing to this regulation.

A few genes encoding TCA cycle enzymes, including *Idh3a* and *Ogdh*, which participate in multienzyme complexes that have been implicated as rate-limiting, were directly targeted by the ZFP36 family and showed increased mRNA stability (Supplementary Fig. S3e; Fig. 4). In addition to fuelling the electron transport chain, TCA cycle intermediates contribute to other metabolic processes. These include the malate-aspartate shuttle, which is important for maintaining redox balance in activated T cells (Fig. 4b). Both *Mdh2* and *Got1* 3’UTRs were bound by ZFP36-family RBPs, and the stability of *Got1* transcripts was increased in dKO T cells (Supplementary Fig. S3e; Fig. 4). Furthermore, several genes encoding rate-limiting enzymes required for coenzyme A synthesis or diversion of citrate into lipid biogenesis pathways, including *Acly*, *Pank3*, *Hmgcr* and *Fasn*, displayed increased expression and direct targeting by the ZFP36 family (Fig. 4; Supplementary Fig. S3e). The UAUU motifs at which we observed ZFP36-family binding frequently displayed higher conservation than surrounding regions of the 3’UTR (Supplementary Fig. S3e), suggesting functional conservation during evolution.

Consistent with the overall redundancy observed between ZFP36 and ZFP36L1 (Supplementary Fig. S1c-e), we found that the majority of genes related to metabolism that were implicated as direct ZFP36-family targets showed remarkable redundancy, with many showing little or no increase in either of the single KO models (Supplementary Fig. S3f.). For almost every gene examined, the increased expression in the dKO was greater than would be expected from an additive effect of the two single KOs. Nonetheless, for some genes including *Hif1a* and *Mthfd2* there was some increase in expression in single KO models, with the greatest effects seen upon deletion of *Zfp36l1*.

It is clear that many of the direct targets of the ZFP36 family encode metabolite transporters or enzymes with a known regulatory role in metabolism, with several implicated as rate-limiting in different contexts. To examine whether this represented an overall preference for targeting rate-limiting genes, we divided the genes involved in several metabolic pathways based on whether they were implicated as rate-limiting through literature searches (Supplementary Table S9). We compared these to control gene sets, not restricted to metabolic genes but with matched expression and 3’UTR length. Genes implicated as rate-limiting were enriched relative to matched controls for direct ZFP36-family targets (Fig. 5g) and for the presence of the UAUUUAUU motif within their 3’UTRs (Fig. 5h), albeit notable exceptions include *G6pdx* and *Phgdh*, whilst other metabolic genes were not. This was accompanied by a trend towards a stronger increase in gene expression in dKO CD4^+^ cells, which reached significance for total mRNA abundance (Fig. 5i). While it is possible that in the context of activated CD4^+^ T cells some of these genes may not be rate-limiting and there are many additional layers of regulation to consider, these data suggest that the ZFP36-family RBPs may preferentially target critical steps in metabolism, with the potential to influence CD4^+^ T cell activation and differentiation.

### Zfp36 and Zfp36l1 limit glutamine metabolism

To identify the downstream effects of ZFP36 family-mediated gene regulation on the metabolic state of CD4^+^ T cells, we used hydrophilic interaction and reversed-phase liquid chromatography mass spectrometry (LC–MS) to quantify the abundance of 52 metabolites in *Zfp36/Zfp36l1* dKO and control CD4^+^ T cells 24 h following activation (Supplementary Table S10). In addition, we performed glucose and glutamine tracing by activating CD4^+^ T cells for 24 h in the presence of ^13^C-labelled glucose or glutamine, followed by gas chromatography mass spectrometry (Supplementary Table S11).

**Figure 6:**
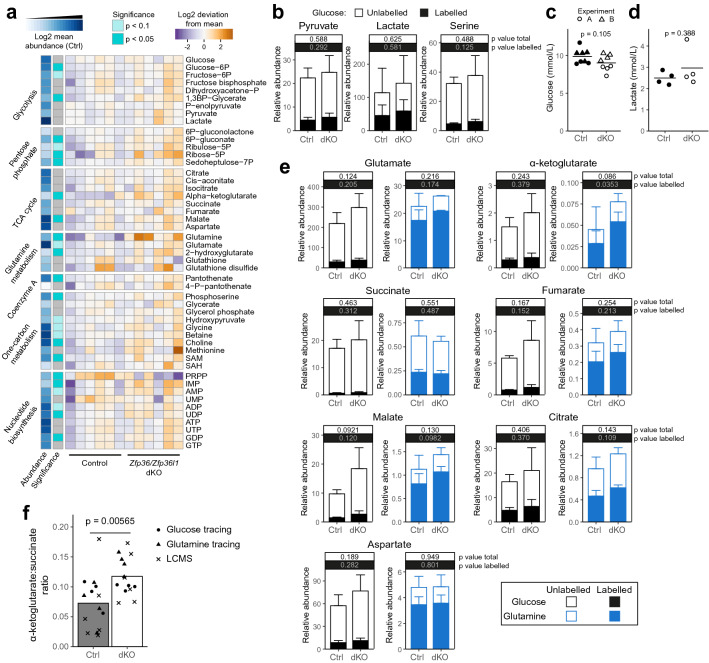
*Zfp36* and *Zfp36l1* limit glutamine metabolism. (**a**) Heatmap showing the abundance of metabolic intermediates from multiple pathways in control and *Zfp36/Zfp36l1* dKO CD4^+^ T cells following 24 h activation, measured by LC–MS. Significantly altered metabolites are indicated, together with the log2-transformed mean abundance of each metabolite in control cells. (**b, e**) Relative abundance of ^13^C-labelled (relative ion counts × fractional labelling) and unlabelled (relative ion counts) metabolites following 24 h activation of control and dKO CD4^+^ T cells in the presence of ^13^C-labelled glucose or glutamine. Bars indicate the mean; error bars indicate the mean + SD. Note that ion counts were normalised to an internal standard within each experiment and are thus not comparable between glucose and glutamine tracing experiments. (**c**, **d**) Abundance of glucose (**c**) and lactate (**d**) measured in culture supernatants following 24 h activation of control and dKO CD4^+^ T cells. In **c**, the shape of the points indicates two independent experiments. (**f**) Ratio of α-ketoglutarate to succinate for samples in each of the metabolomics datasets. P values for (**a**-**f**) were calculated using unpaired Student’s t-tests.

Most glycolytic intermediates showed little change in abundance by LC–MS (Fig. 6a), and there was no significant difference in glucose carbon incorporation into pyruvate, lactate or serine (Fig. 6b). Accordingly, the amount of glucose remaining and lactate accumulation in the media were comparable between genotypes (Fig. 6c,d). We observed a significant increase in the abundance of several metabolites from the pentose phosphate pathway by LC–MS (Fig. 6a), including ribose-5-phosphate and sedoheptulose-7-phosphate, suggesting that in the absence of ZFP36 and ZFP36L1, an increased amount of glucose might be diverted, via glucose-6-phosphate, into this pathway (Fig. 3b). An increased abundance of S-adenosyl methionine (SAM) (Fig. 6a), correlating with the increase in *Mat2a* mRNA abundance, is suggestive of increased activity of the methionine cycle.

Of all metabolites measured by LC–MS, glutamine displayed the largest increase in abundance, being over seven-fold higher on average in dKO compared with control CD4^+^ T cells (Fig. 6a; Supplementary Fig. S4a; Supplementary Table S10). Glutamine oxidation in the TCA cycle through conversion via glutamate to α-ketoglutarate (Fig. 4b) is critical during T cell activation^3^. Glutamate can also be converted to α-ketoglutarate through the activity of transaminases such as GOT1/2 in the malate-aspartate shuttle or PSAT1 during serine biosynthesis. We observed a trend towards increased glutamate and a three-fold increase in α-ketoglutarate, accompanied by a trend towards increased abundance of several other TCA intermediates including citrate, isocitrate and malate (Fig. 6a; Supplementary Fig. S4a), consistent with increased glutaminolysis in the dKO. Glutamine tracing supported it to be the principal contributor to increased glutamate, α-ketoglutarate, malate, fumarate and citrate, with glucose incorporation into these intermediates comparable between control and dKO cells (Fig. 6e). Whilst we were unable to distinguish the L- and D- enantiomers, 2-hydroxyglutarate was increased (Fig. 6a). The increased glutamine labelling of TCA intermediates, with the exception of α-ketoglutarate, was attributed to the M + 4 species, whilst the M + 3 and M + 5 species were low and comparable between genotypes (Supplementary Fig. S4b). This suggests that reductive carboxylation of α-ketoglutarate to form citrate, a mechanism utilised in hypoxia that can result in D-2-hydroxyglutarate^74,75^, was not increased. The ratio of α-ketoglutarate to succinate, which is known to impact differentiation potential^76–78^, was increased in dKO cells (Fig. 6f). These results, together with our transcriptomics, suggest that regulation of *Gls*, *Glud1* and *Slc38a2* by ZFP36 and ZFP36L1, through direct and/or indirect means, limits glutamine metabolism which may affect the course of differentiation of the activated CD4^+^ T cell.

### ZFP36 and ZFP36L1 limit glutamine-dependent CD4^+^ T cell differentiation

**Figure 7:**
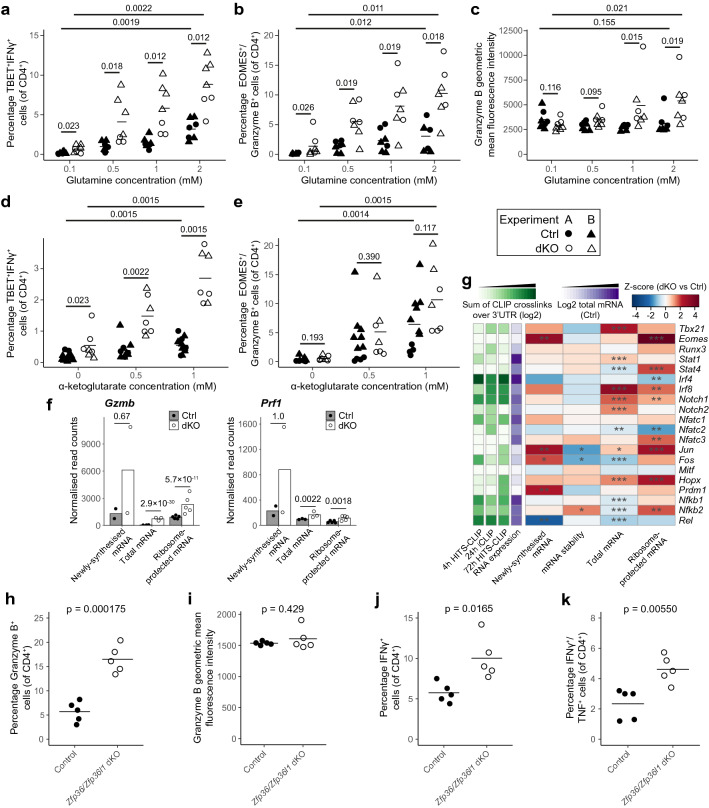
ZFP36 and ZFP36L1 limit CD4^+^ T cell differentiation into Th1 and cytotoxic-like phenotypes. (**a, d**) Percentage of TBET^+^IFNγ^+^ control and dKO CD4^+^ T cells after 24 h activation with anti-CD3 and anti-CD28 in Th1-polarising conditions, followed by 48 h maintenance in IL-2. Throughout, the media was supplemented with normal (2 mM) or limiting glutamine (**a**) or was glutamine-deprived but supplemented with cell permeable esterified α-ketoglutarate (**d**), as indicated. Representative flow cytometry plots are shown in Supplementary Fig. S5a and c. (**b-c, e**) Percentage of EOMES^+^ Granzyme B^+^ (**b, e**), and mean fluorescence intensity of Granzyme B (**c**) in control and dKO cells following Th1 differentiation as in **a** and **d**. Representative flow cytometry plots are shown in Supplementary Fig. S5b and d. *P* values in **a-e** were calculated using a Kruskal–Wallis rank sum test and FDR corrected. (**f**) DESeq2 normalised read counts in control and dKO mice, and FDR-adjusted p values for *Gzmb* and *Prf1*. (**g**) Heatmap showing z-scores for changes in transcriptomic data for genes encoding transcription factors of interest. The log2-transformed sum of crosslinks (with FDR < 0.25) over the 3’UTR for each CLIP dataset, normalised to the 90% quantile for detected genes, and mean mRNA abundance for control samples are displayed. Significance from DESeq2 analysis (or as described in methods for stability) is indicated: *** FDR-adjusted p value < 0.05; ** raw p value < 0.05; * raw p value < 0.1. (**h**–**k**) Percentage of CD4^+^ T cells isolated from the lung of control and dKO mice 10 days following infection with influenza A that express Granzyme B (**h**), IFNγ (**j**) or IFNγ and TNF (**k**); and mean fluorescence intensity of Granzyme B (**i**). Data are from a single experiment, representative of 2 (**k**) or 3 (**h-j**) independent experiments; p values were calculated using an unpaired Student’s t-test. Representative flow cytometry plots are shown in Supplementary Fig. S5f,g.

Glutamine metabolism has been implicated in CD4^+^ T cell differentiation, with studies utilising glutamine deprivation and supplementation of α-ketoglutarate suggesting that glutamine promotes Th1 differentiation^9,77^, whilst deletion of *Gls* or use of the inhibitor CB839 suggested the opposite^8^. This prompted us to examine how the in vitro differentiation of CD4^+^ T cells under conditions of controlled glutamine availability was influenced by the absence of ZFP36 and ZFP36L1. We activated naïve CD4^+^ T cells in conditions that promote the acquisition of a Th1-like phenotype for 24 h, and supplemented the cells with IL-2 to promote their survival for an additional 48 h. The accumulation of TBET^+^IFNγ^+^ cells was dependent on glutamine, since reducing its concentration from 2 to 0.1 mM resulted in vastly reduced frequencies of TBET^+^IFNγ^+^ cells in both control and dKO mice (Fig. 7a; Supplementary Fig. S5a). The frequency of TBET^+^IFNγ^+^ cells was enhanced in the dKO mice compared to the control mice, and the effect was apparent even at the lowest glutamine concentration tested. The advantage of dKO over control cells increased with increasing glutamine availability, from an average gain of 0.5% TBET^+^IFNγ^+^ cells at 0.1 mM to 5.7% at 2 mM, clearly highlighting a role for increased glutamine uptake and/or metabolism in supporting this. Furthermore, at all glutamine concentrations, differentiation of the dKO was enhanced, indicating the existence of glutamine-independent mechanisms. CD4^+^ T cells can acquire a cytotoxic phenotype, marked by their ability to produce granzyme B^79^. In the differentiation cultures, the expression of granzyme B together with the transcription factor EOMES demonstrated a similar pattern of glutamine dependence to TBET and IFNγ, as did the mean fluorescence intensity of granzyme B (Fig. 7b,c; Supplementary Fig. S5b). Thus, acquisition of the cytotoxic CD4^+^ T cell phenotype is limited by ZFP36/ZFP36L1 and the availability of glutamine.

### α-Ketoglutarate promotes the acquisition of markers of the cytotoxic Th1 phenotype

To establish whether supplementation with α-ketoglutarate would promote the differentiation of cells under conditions of glutamine restriction, we added cell-permeable esterified α-ketoglutarate to glutamine-deprived cultures. This promoted the differentiation of both control and dKO cells towards both Th1 and cytotoxic phenotypes (Fig. 7d,e; Supplementary Fig. S5c-d). Similar to glutamine, α-ketoglutarate selectively enhanced the differentiation of dKO cells towards TBET^+^IFNγ^+^ cells compared to controls (Fig. 7d). Enhanced acquisition of the cytotoxic phenotype was also apparent (Fig. 7e). This suggests the potential for an increased capacity to use this metabolite to support the differentiation advantage of dKO cells. In addition, differentiation towards the Th1 cell fate was higher in dKO compared with control CD4^+^ T cells irrespective of exogenous α-ketoglutarate concentration. Whilst enhanced utilisation of glutamine and/or α-ketoglutarate likely contributes to increased differentiation, our data also indicate roles for ZFP36/ZFP36L1 in differentiation that are independent of glutaminolysis.

### ZFP36 and ZFP36L1 regulate transcription factors and effector molecules linked to cell fate

In our transcriptomic data, we observed increased abundance and translation of mRNA encoding both granzyme B and perforin (Fig. 7f), indicating that activated dKO CD4^+^ T cells exhibit a greater tendency to differentiate towards a cytotoxic CD4^+^ T cell phenotype prior to the onset of cell division. Moreover, mRNA abundance and/or translation were increased for genes encoding TBET (*Tbx21*), EOMES and HOPX, transcription factors that promote this cell fate (Fig. 7g). This was accompanied by ZFP36 family binding to the 3’UTRs of *Hopx* and *Tbx21* transcripts, as well as to *Gzmb* (Supplementary Fig. S5e). Other transcription factors linked to cytotoxic CD4^+^ T cell fate, such as BLIMP1 (*Prdm1*), RUNX3 and IRF4, were not significantly increased, and translation of *Irf4* was decreased despite strong evidence for binding of its mRNA by the ZFP36 family (Fig. 7g). Given our previous identification of transcription factors regulated by the ZFP36 family during CD8^+^ T cell differentiation^51^, we also examined these transcripts in CD4^+^ T cells. Both *Notch1* and *Irf8* showed increased mRNA abundance and translation, accompanied by ZFP36-family binding in their 3’UTR, whilst *Nfkb2* was bound and increased in stability and translation of its mRNA (Fig. 7g). By contrast, *Nfkb1* and *Rel* were not increased 24 h following activation, despite ZFP36-family binding in their 3’UTRs. These data indicate that whilst the ZFP36 family may regulate both CD4^+^ and CD8^+^ T cell differentiation through targeting some of the same transcription factors, there might also be context-specific effects which differ between these cell types. These data suggest that transcription factors that influence the differentiation of CD4^+^ T cells and effector molecules are repressed by ZFP36 and ZFP36L1.

### ZFP36 and ZFP36L1 limit the accumulation of cytotoxic CD4^+^ T cells in vivo

To determine whether ZFP36/ZFP36L1 were limiting factors for cytotoxic CD4^+^ T cells in vivo, we infected *Zfp36*^fl/fl^/*Zfp36l1*^fl/fl^*Cd4*^cre^ mice with influenza A virus (PR8 H1N1) and measured CD4^+^ T cells in the lung 10 days following infection. There was an almost three-fold higher frequency of granzyme B expressing CD4^+^ T cells (Fig. 7h,i; Supplementary Fig. S5f.). This was accompanied by an almost two-fold increase in cells expressing IFNγ and TNF (Fig. 7j,k; Supplementary Fig. S5g). This is consistent with our in vitro differentiation data, suggesting that the ZFP36 family limits the production of cytokines and effector molecules typical of Th1 and cytotoxic CD4^+^ T cells during an in vivo immune response.

## Discussion

Through the application of a transcriptome-centric multiomics approach, we found evidence that the ZFP36 family of RBPs has a central role in orchestrating metabolic reprogramming and differentiation following the activation of naïve CD4^+^ T cells. We discovered genes and pathways implicated as direct and indirect ZFP36/ZFP36L1 targets and revealed the mechanisms they likely employ to affect gene expression. The data support the existence of post-transcriptional operons in activated T cells that coordinate the expression of genes within pathways^80^. Furthermore, they suggest extensive integration between pathways mediated by the actions of the ZFP36 family through direct effects on mRNA stability and translation and indirect regulation of transcription, likely via direct effects on transcription factors and/or epigenetic regulators.

The complexity of the roles of the ZFP36 family in gene expression regulation is exemplified by the different modalities used to regulate cytokine gene expression in T cells. In our data, *Ifng*, *Ccl3* and *Ccl4* were regulated by mRNA stability, whilst *Tnf* and *Il2* were regulated translationally. For *Tnf*, this is consistent with data from CD4^+^ T cells in a single KO of *Zfp36*, whilst in that context, *Il2* expression was not increased^46^. The results for *Il2* in CD4^+^ T cells are also in contrast to CD8^+^ T cells early following activation, in which a substantial increase in mRNA abundance within dKO cells was driven by increased transcript stability^51^. Furthermore, the same transcript, e.g., *Tnf*, can be regulated in distinct ways depending on the cellular context, as highlighted by Kovarik^81^.

We found examples where clear binding by ZFP36-family RBPs to mRNA, such as *Irf4*, was not accompanied by any evidence for increased transcript stability or translation. The phenomenon of mRNA binding by ZFP36 without apparent consequence for stability or translation has been noted in other contexts^82^, and its significance remains to be determined. These observations reflect context-specific functions of ZFP36-family RBPs, each of which regulate RNA decay and translation^45,46,83^, but emphasise that these actions can be discharged independently of each other^81^. How this is achieved biochemically is unknown, but the extensive phosphorylation of ZFP36 family members, their participation in multiprotein complexes and intracellular compartmentalisation are areas for future research that should provide insights into this question. Indeed, both ZFP36 and ZFP36L1 have been linked to the formation and function of cytoplasmic RNA granules^84^, and in non-lymphoid cells ZFP36L1 has been visualised associated with membrane-less structures, where it is proposed to play a role in localised translation^42^. In these contexts, the function of ZFP36L1 is independent of its effects on the amounts of mRNA or protein. Thus, interactions between ZFP36 family members and target transcripts that did not correlate with changes in the transcriptome may nevertheless have been consequential for the intracellular localisation of proteins. These considerations may also prove relevant to metabolism in which baker’s yeast provides a paradigm with enzymes of glycolysis translated in RNA granules^85^.

Our multiomics approach revealed that, whilst from the transcriptomic data, glycolysis appeared to be increased in the dKO, the abundance of glycolytic intermediates, or glucose incorporation into linked pathways such as the TCA cycle was not increased. This highlights the importance of validating hypotheses that are driven by transcriptomic data, particularly when considering complex and highly interconnected pathways such as anabolic metabolism. We also observed no evidence for engagement of the alternative pyruvate carboxylase-mediated route into the TCA cycle^86^, since *Pcx* expression was low, as was the abundance of M + 3 citrate with labelled glucose. Our data raise the possibility that some glucose might instead be diverted into the pentose phosphate pathway, ultimately contributing to the increased nucleotide synthesis required by rapidly dividing cells following activation, but further experimental evidence will be required to confirm this hypothesis. Glucose might also be diverted into the hexosamine pathway, which also utilises glutamine, providing substrates for the glycosylation required for increased cytokine production in dKO cells, thus protecting against the unfolded protein response^87^. Notably, *Gfpt1*, encoding the rate-limiting enzyme of the hexosamine pathway, is increased in mRNA abundance, stability and translation in dKO cells (Supplementary Tables S2, S4, S5) and was identified as a direct ZFP36-family target in the 72 h HITS-CLIP data (Supplementary Table S7).

In contrast to glucose, glutamine anaplerosis into TCA intermediates was increased, indicative of increased glutaminolysis, although other mechanisms such as transaminase activity might also contribute. A limitation of our study is that the localisation of metabolites is unknown, limiting our ability to distinguish between pathways. Increased differentiation towards Th1 and cytotoxic CD4^+^ T cell fates was dependent on glutamine and partially rescued by cell-permeable esterified α-ketoglutarate; thus, it is likely that increased glutaminolysis in the dKO contributes to enhanced differentiation. Our data also suggest that an increased capacity to use α-ketoglutarate impacts on differentiation, potentially via its modulation of downstream metabolic pathways or oxygen-dependent epigenetic regulators^12,77,78,88^. However, in all conditions, differentiation in the dKO was maintained at a higher level compared with controls, indicating that altered glutaminolysis or α-ketoglutarate abundance is insufficient to explain this phenotype. The nature of the additional pathway or process regulated by the ZFP36 family, which limits differentiation independently of glutamine, is not known, although our data suggest that regulation of other metabolites or transcription factors may contribute. It is also likely that regulation of cytokines by the ZFP36 family, including increased production of IFNγ and IL-2 by dKO cells, impacts on differentiation, although the repression of multiple, potentially antagonistic, cytokines by ZFP36 and ZFP36L1 make the effects of such regulation hard to predict. Furthermore, there is potential for direct epigenetic regulation mediated by ZFP36 and ZFP36L1 via their actions on histone-lysine demethylases, as has been shown for ZFP36L2 in mouse oocytes^89^. *Mthfd2* and *Mat2a,* which we identified as prominent ZFP36-family bound targets, are implicated as metabolic checkpoints in CD4^+^ T cells, with their inhibition resulting in reduced differentiation towards the Th1 lineage^27,73^. *Mthfd2* was strongly increased in stability and translation in dKO cells, and whilst *Mat2a* was not, perhaps suggestive of an indirect effect, the clear 3’UTR binding to a conserved ARE is suggestive of regulation. It remains possible that direct effects of ZFP36-family targeting might be evident prior to the 24 h timepoint at which our transcriptomic experiments were performed. Elucidation of the mechanisms that are integrated by ZFP36/ZFP36L1 to restrain Th1 and cytotoxic CD4^+^ T cell differentiation will be an important area for future study.

Our data highlight parallels between ZFP36/ZFP36L1-mediated regulation of differentiation in CD4^+^ and CD8^+^ T cells, in particular with CD4^+^ T cells being driven towards a cytotoxic phenotype. Indeed, some of the transcription factors identified as regulated in our previous study on CD8^+^ T cells^51^ were also bound and increased in expression in dKO CD4^+^ T cells, although expression of others such as *Nfkb1* and *Rel* was unaltered, indicative of context-dependent regulation. We have also observed ZFP36L1 binding to some of the transcripts identified here in the CD8^+^ iCLIP data generated in that study, including *Gls*, *Glud1*, *Mthfd2*, *Mat2a* and *Pfkp*. The implications of this binding for the metabolism of CD8^+^ T cells, and how this impacts differentiation, provides another avenue for future research.

## Conclusions

Regulation of the metabolic state of CD4^+^ T cells is critically important for their differentiation and function. Many interconnected pathways must be intricately controlled to ensure appropriate nutrient availability for cell division and energy production, as well as providing substrates, such as α-ketoglutarate and S-adenosyl-L-methionine, required for methylation of DNA, RNA and protein, with wide-reaching consequences for gene regulation and cell fate. Metabolic reprogramming is well known to be promoted and integrated by transcription factors such as cMYC and HIF1α. Our data implicate ZFP36 and ZFP36L1 as providing a further layer of post-transcriptional regulation, and suggest that direct regulation of several key metabolic genes is reinforced by targeting of the transcription factors themselves and consequently limits differentiation towards the cytotoxic Th1 cell fate. Dissecting the relative importance of these direct and indirect mechanisms, and whether this changes at different times following ZFP36 and ZFP36L1 induction, will be an important next step in understanding regulation by these RBPs. Additional experiments, such as engineering of the 3’UTRs of proposed direct target genes, will also give further mechanistic insight. Their involvement in the regulation of genes involved in numerous different metabolic pathways highlights the ZFP36 family as a promising candidate for coordinating between these pathways, ultimately ensuring an appropriate immune response upon encountering pathogens.

## Methods

### Mice

The *Zfp36*^tm1.1Tnr^ (MGI:7,325,470) and *Zfp36l1*^tm1.1Tnr^ (MGI:4,819,154)^44,90^ targeted conditional alleles; and Cre strains Tg(Cd4-cre)1^Cwi^ (MGI: 2,386,448; J:73,127)^91^ and Tg(CD2-icre)4^Kio^ (MGI:2,449,947)^92^ have been previously described.. Mice were bred and maintained in the Babraham Institute Biological Support Unit under Specific Opportunistic Pathogen Free (SOPF) conditions. After weaning, mice were transferred to individually ventilated cages with 1–5 mice per cage. Mice were fed the CRM (P) VP diet (Special Diet Services) ad libitum and received environmental enrichment. Healthy mice aged between 7 and 18 weeks of both sexes were used for experiments unless noted otherwise; littermate controls with floxed alleles were used when making comparisons. Due to the availability of mice, sexes were not always balanced between genotypes; see method details for individual experiments. For this reason, Y chromosome genes were excluded from transcriptomic analyses. Except for transcriptomics and iCLIP where mice were pooled as detailed in the relevant methods sections, all experiments were performed on individual mice. No mice were excluded; data points were not excluded except in tracing experiments as indicated below. Where it was not possible to process all mice/samples in parallel, at least one sample of each genotype was processed together, and genotypes were alternated for data acquisition. Except for the influenza infection, experiments were not blinded. Procedures were approved by the Babraham Institute Animal Welfare, Experimentation and Ethics Committee and licenced under the Animals (Scientific procedures) Act 1986 and EU Directive 2010/63/EU; all experiments were performed in accordance with these guidelines and regulations. Experiments in this study are reported in accordance with ARRIVE guidelines^93^.

### T cell purification and stimulation in non-skewing conditions

Naïve CD4^+^ T cells were isolated from spleen, mesenteric and peripheral lymph nodes using the Miltenyi Biotech CD4^+^ CD62L^+^ T Cell Isolation Kit (Miltenyi 130–106-643) and purity was checked by flow cytometry analysis. T cells were resuspended at a final concentration of 500,000 cells/ml, cultured in RPMI 1640 containing 10% fetal calf serum (FCS), 50 µM β–mercaptoethanol, 2 mM glutamine and 100 U/ml penicillin/streptomycin, and activated through crosslinking of CD3ε by precoating tissue culture plates overnight at 4 °C with 2C11 monoclonal antibody (Bio X Cell, 10 µg/ml; RRID:AB_1107634), together with the 37.51 anti-CD28 monoclonal antibody as a hybridoma supernatant or purified antibody (Bio X Cell; RRID:AB_1107624) at 10 µg/ml, unless otherwise indicated in the methods or figure legends.

### In vitro differentiation assays

Naïve CD4^+^ T cells were isolated and activated for 24 h (100,000 cells in 200 µl per sample) in Th1-skewing conditions: 96-well flat bottom plates were precoated overnight at 4 °C with 5 µg/ml anti-CD3 (clone 2C11, Bio X Cell; RRID:AB_1107634) and 1 µg/ml anti-CD28 (clone 37.51, Bio X Cell; RRID: AB_1107624); cells were cultured in RPMI 1640 lacking glutamine and phenol red (ThermoFisher 32,404,014) and supplemented with 10% FCS, 50 µM β–mercaptoethanol, 100 U/ml penicillin/streptomycin and varying concentrations (0.1–2 mM) of GlutaMAX (ThermoFisher 35,050,061), in addition to 5 µg/ml anti-IL4 (BioLegend 504,122), 20 ng/ml IL-2 (Peprotech 212–12), 15 ng/ml IL12 (Peprotech 210–12) and 1 ng/ml IL7 (Peprotech 217–17). A Th0 control was also included, with 2 mM GlutaMAX and excluding anti-IL4, IL7 and IL12. For the esterified α-ketoglutarate titration, no GlutaMAX was added, but instead media was supplemented with varying concentrations of the cell permeable esterified α-ketoglutarate, dimethyl 2-oxoglutarate (Sigma 349631). Following 24 h activation in skewing conditions, 50 µl from each culture was taken and assessed by flow cytometry to ensure equivalent levels of activation. The remaining cells were transferred to fresh plates without anti-CD3 or anti-CD28 coating and maintained for a further 48 h in media as above, excluding anti-IL4, IL7 and IL12, using the same concentrations of GlutaMAX/dimethyl 2-oxoglutarate as for the first 24 h. Cells were then harvested for analysis by flow cytometry. For glutamine titrations, two independent experiments were performed, one including three and the other four biological replicates per genotype. For 2-oxoglutarate titrations, two independent experiments were performed; the first included four biological replicates per genotype, and the second included seven control and three dKO biological replicates. In all experiments, two technical replicates for each of the biological replicates were performed; these were averaged.

### Cytokine measurements

For measurements of IFNγ, TNF and IL-2 in comparison with RNA measurements over a 96 h time course, cytokine proteins were quantified using a BD cytometric bead array (CBA) from BD Biosciences and analysed by flow cytometry according to the manufacturer’s protocol. RNA was quantified using QuantiTect primer assays from Qiagen (QT01038821 and QT00104006 for mouse *Ifng* and *Tnf*, respectively) according to the manufacturer’s instructions. Relative transcript abundances were normalised to *B2m.*

### TaqMan assays for ZFP36 family genes

For TaqMan assays Platinum Taq Supermix with UDG (Invitrogen) was used with commercial TaqMan probes and primers (*Zfp36*; ABI Mm00457144_m1) or custom probes and primers for *Zfp36l1* (F: CTTCACGACACACCAGATCCTAGT; R: TGCTGTAGTTGAGCATCTTGTTACC; Probe: AACGCCCACGATGA) and *Zfp36l2* (F: ATGTCGACCACACTTCTGTCACC; R: CTTCTTGTCCAGCATGTTGTTCAG; Probe: AGGGATTTCTCCGTCTTGC), as described previously^94^. 5 μl of cDNA sample was used in a 25 μl total reaction volume. qPCR was carried out using a CFX96 (Bio-Rad) with an annealing/extension temperature of 60 °C. Relative transcript abundances were normalised to *B2m.*

### Flow cytometry

All antibodies, fluorochromes and suppliers used are listed in Supplementary Table S12. Single-cell suspensions were prepared in phosphate buffered saline (PBS) containing 1% FCS and 1 mM EDTA. For surface staining, cells were incubated first with Fc-block (24G2) in buffer for 15 min and then with specific antibodies for 20–30 min at 4 °C in the dark. For intracellular staining, cells were first surface stained and then either fixed and permeabilised with the BD Cytofix/Cytoperm kit, followed by staining in BD Permwash containing 0.5% FCS for 1 h at 4 °C in the dark (influenza experiments), or fixed and permeabilised using the eBioscience Foxp3 staining kit and stained overnight at 4 °C in eBioscience Permwash containing Fc-block (24G2) (in vitro Th1 differentiation experiments). Data were acquired using a Fortessa flow cytometer equipped with 355 nm, 405 nm, 488 nm, 561 nm and 640 nm lasers (Beckton Dickinson; RRID:SCR_019601), and analysed using FlowJo software (version 10.6 or 10.8).

### Influenza infection

8–12-week-old female mice were used for all experiments. During infection, animals were anesthetised with isoflurane and sub-lethally infected in a blinded fashion by intranasal administration of 10 PFU of influenza A/Puerto Rico/8/34 (PR8 H1N1). Mice were euthanised 10 days post-infection, without blinding, and the lungs were perfused by injecting 5 ml of HBSS + 1 mM EDTA and 25 mM HEPES through the right ventricle of the heart. Single-cell suspensions from the lungs were prepared with collagenase A (Sigma-Aldrich) 0.5 mg/ml and DNAse I (Sigma- Aldrich) 0.15 KU/ml in sterile HBSS with 5% FBS for 1 h at 37 °C. The lung digest was filtered, and mononuclear cells were resuspended in 1% BSA and 1 mM EDTA containing PBS (FACS Buffer). Viable cells were counted using trypan blue exclusion and stained for flow cytometry as described above. Two independent experiments including all flow cytometry markers each included five biological replicates; the third independent experiment which omitted TNF staining included four biological replicates.

### mRNA sequencing

mRNA-seq libraries were generated for control (*Zfp36*^fl/fl^*Zfp36l1*^fl/fl^) and dKO (*Zfp36*^fl/fl^*Zfp36l1*^fl/fl^*Cd4*^cre^) CD4^+^ T cells following activation of purified naïve cells for 24 h, in a total of three biological replicates. Each replicate comprised a pool of two mice aged 7–8 weeks old, with varying representation of males and females. Following activation, RNA was isolated using TRIzol (Invitrogen), and libraries were prepared using the TruSeq Stranded mRNA Sample Prep Kit (Illumina) before sequencing on the Illumina HiSeq 2000 (100 bp single end). For mRNA-seq libraries from single KO CD4^+^ T cells (*Zfp36*^fl/fl^*Cd4*^cre^ and *Zfp36l1*^fl/fl^*Cd2*^cre^) and corresponding floxed controls, naïve cells were activated and RNA isolated as above, before library preparation using the RNA TruSeq V2 mRNA kit (Illumina) and sequencing on the Illumina HiSeq 2500 (50 bp paired end).

### Metabolic labelling of RNA and generation of sequencing libraries from RNA fractions

Metabolic labelling with 4-thiouridine was carried out essentially as previously described^95^, with two biological replicates for control (*Zfp36*^fl/fl^*Zfp36l1*^fl/fl^) and dKO (*Zfp36*^fl/fl^*Zfp36l1*^fl/fl^*Cd4*^cre^). The first replicate for control and dKO were processed in a separate batch from the second replicate. Naïve CD4^+^ T cells pooled from 10 to 16 mice aged 7–18 weeks (mix of male and female) were activated in vitro for 24 h, with 4-thiouridine (Sigma, T4509) added to a final concentration of 500 μM for the last hour. RNA was purified with TRIzol (Invitrogen), before labelling 100–160 µg with Biotin-HPDP (Pierce, 50 mg EZ-Link Biotin-HPDP) in 100 mM Tris pH 7.4/10 mM EDTA at room temperature for 1.5 h with rotation. RNA was separated from unincorporated biotin-HPDP by chloroform/isoamyl-alcohol (24:1) extraction and ethanol precipitation, resuspended in TE and the labelled (newly-synthesised) and unlabelled (pre-existing) RNA were separated using streptavidin-coated magnetic beads. The newly-synthesised and pre-existing RNA fractions, in addition to the total RNA, were used to generate libraries for RNA sequencing using the TruSeq Stranded mRNA Sample Prep Kit (Illumina). Libraries were sequenced on an Illumina HiSeq 2000, with 100 bp single-end reads.

### Ribosome profiling

Ribosome profiling (Ribo-Seq) libraries were generated from five biological replicates for control (*Zfp36*^fl/fl^*Zfp36l1*^fl/fl^) and dKO (*Zfp36*^fl/fl^*Zfp36l1*^fl/fl^*Cd4*^cre^) mice. Replicates 1 and 2 for control and dKO were processed in a separate batch from replicates 3–5. Mice were aged 10–13 weeks; for controls, each replicate comprised an individual male mouse, whilst for dKO, each replicate was a pool of two mice, one male and one female. Libraries were prepared using the ART-seq Ribosome Profiling kit (Epicentre, Illumina) according to the manufacturer’s instructions. Following 24 h activation of naïve CD4^+^ T cells, cells were treated with cycloheximide (CHX, 100 µg/ml) three minutes before rapid cooling of the culturing plate, cell extract preparation, isolation of ribosome-protected fragments and library generation. Libraries were sequenced on an Illumina HiSeq 2000, on a paired-end 50 bp run, but only the first read was used since this is sufficient to capture the full ribosome-protected fragment.

### iCLIP

iCLIP was performed on 24 h-activated WT CD4^+^ T cell lysates as previously described^96^ using anti-BRF1/2 (1 µg/IP; Cell Signaling 2119; RRID: AB_10695874) or anti-Tis11b (4 µg/IP; Abcam ab42473; RRID: AB_883662) antibodies, with three replicates for each antibody. For each replicate, naïve T cells were pooled from three mice aged 9–16 weeks prior to activation. For one replicate with the BRF1/2 antibody, low- and high-molecular-weight protein:RNA complexes were isolated separately for library generation, but data were pooled for analysis. Control samples from CD4^+^ T cells lacking ZFP36L1 (*Zfp36l1*^fl/fl^*Cd4*^cre^) were included in the experiment to validate the specific pulldown of ZFP36L1 protein:RNA complexes but were not analysed further. Libraries were sequenced on a HiSeq 2500 RapidRun (50 bp single-end).

### Metabolic profiling with liquid chromatography mass spectrometry (LC–MS)

LC–MS was performed on six biological replicates for control and dKO mice, each comprising a single mouse. Naïve CD4^+^ T cells (5 × 10^5^ cells/ml) were activated for 24 h in vitro with anti-CD3 and anti-CD28 antibodies (10 µg/ml each) before harvesting the cells, resulting in a total of 2.5–3 million cells per replicate. Cells were washed with 1 × PBS before snap freezing on dry ice.

#### Extraction of aqueous metabolites

Cell pellets were washed with PBS and then subjected to extraction using the methanol:chloroform method described by Folch^97^. Briefly, 1 ml of 2:1 chloroform:methanol was added to the Eppendorf tube containing the washed cell pellet followed by 400 µl of ice-cold water, and the samples were thoroughly vortexed and sonicated for 5 min before centrifugation at 21,000 g for 5 min. After centrifugation, the aqueous (top layer) and organic (bottom layer) fractions were separated and aliquoted into separate screw-cap tubes, which were both kept on dry ice. A further 1 ml of 2:1 chloroform:methanol was added to the original tube containing the protein pellet, and the extraction was repeated as described above. The resulting layers were dried using a centrifugal evaporator (Savant, Thermo Fisher) and stored at -20 °C prior to further preparation and analysis.

#### LC–MS sample preparation

Dried aqueous extracts of cells were reconstituted in 100 µl of 7:3 acetonitrile:0.1 M aqueous ammonium carbonate water containing 2 µM [^13^C_10_, ^15^N_5_] adenosine monophosphate and adenosine triphosphate [^13^C_10_, ^15^N_5_], 10 µM succinic acid ^13^C_10_ and 1 in 5000 diluted [U^13^C_10_, U^15^N_5_] mixture of amino acids (all from Sigma Aldrich) as internal standards. The resulting solution was vortexed and then sonicated for 5 min followed by centrifugation at 21,000 g to pellet any remaining undissolved material. After centrifugation, the supernatant was transferred with an automatic pipette into a 300 µl vial with insert (Fisher Scientific) and capped (Agilent) ready for analysis.

#### LC–MS analysis of aqueous metabolites

For MS analysis, a Q Exactive Plus orbitrap (RRID: SCR_020552) coupled to a Vanquish Horizon ultra-high-performance liquid chromatography system was used. Samples were then analysed using a bridged ethylene hybrid (BEH) amide hydrophilic interaction liquid chromatography (HILIC) approach for the highly polar aqueous metabolites. For this analysis, the strong mobile phase (A) was 100 mM ammonium carbonate, and the weak mobile phase was acetonitrile (B) with 1:1 water:acetonitrile used for the needle wash. The LC column used was a BEH amide column (150 × 2.1 mm, 1.7 µm, Waters). The following linear gradient was used: 20% A in acetonitrile for 1.5 min followed by an increase to 60% A over 2.5 min with a further 1 min at 60% A, after which the column was re-equilibrated for 1.9 min. The total run time was 7 min, the flow rate was 0.6 ml/min, and the injection volume was 5 µl. After HILIC analysis, samples were dried and reconstituted in the same volume of 10 mM ammonium acetate prior to orthogonal mixed mode analysis using an ACE Excel C18-PFP column (150 × 2.1 mm, 2.0 µm, Hichrom). Mobile phase A consisted of water with 0.1% formic acid, and mobile phase B consisted of acetonitrile with 0.1% formic acid. For gradient elution, mobile phase B was held at 0% for 1.6 min followed by a linear gradient to 30% B over 4.0 min, a further increase to 90% over 1 min and a hold at 90% B for 1 min with re-equilibration for 1.5 min, giving a total run time of 6.5 min. The flow rate was 0.5 ml/min, and the injection volume was 3.5 µl. The needle wash used was 1:1 water:acetonitrile. The source parameters used for the Q Exactive Plus were a vapouriser temperature of 400 °C, an ion transfer tube temperature of 300 °C, an ion spray voltage of 3.5 kV (2.5 kV for negative ion mode) and a sheath gas, auxiliary gas and sweep gas of 55, 15 and 3 arbitrary units, respectively, with an S-lens RF (radio frequency) of 60%. A full scan of 60–900 m*/z* was used at a resolution of 70,000 ppm, where positive and negative ion mode assays were run separately to maximise data points across a peak at the chosen resolution. For the analysis of CoA and other higher mass species, a unique mass spectrometry methodology was employed where the full scan mass range was reduced to 600–1000 m*/z*, the capillary temperature was increased to 350 °C and the S-lens RF to 100%.

#### LC–MS data processing

Data were acquired, processed and integrated using Xcalibur (Version 4.1, Thermo Fisher Scientific) and Compound Discoverer (Version 3.1, Thermo Fisher Scientific). Metabolites of interest were identified using high-resolution *m/z* values as specified in the METLIN database (Scripps Research Institute) corresponding to their [M + H]^+^ or [M-H]^-^ ion adducts in positive or negative ionisation modes, respectively. Compound retention times were validated against known external standards. Peak areas corresponding to metabolite levels were manually quantified and normalised to internal standard and total ion content (as appropriate) and presented as relative areas. All sample data were processed using Compound Discoverer (Version 3.1, Thermo Fisher Scientific) to accurately calculate the total ion content for normalisation. Data were collated using Excel (Microsoft, Windows 10) and normalised to the total ion content for each sample.

Heatmaps for visualisation of metabolite abundance were plotted as described for transcriptomic data below.

### Glucose and glutamine tracing

For tracing experiments, 3 × 10^6^ cells per condition (7 × 10^5^ cells/ml), with a total of four biological replicates for each genotype/condition, were activated for 24 h with 10 µg/ml plate-bound anti-CD3 and 10 µg/ml plate-bound anti-CD28 antibodies in media containing 10% FCS, 50 µM β–mercaptoethanol, 100 U/ml penicillin/streptomycin (Gibco 15,140) and 2 mM GlutaMAX. Glucose tracing was performed using SILAC RPMI medium (Gibco A2494201) supplemented with 10 mM D-Glucose-^13^C_6_ (Sigma 389,374), whilst glutamine tracing was performed using RPMI medium (Gibco 32,404,014) supplemented with 2 mM ^13^C_5_ Glutamine (Cambridge Isotope Laboratories Inc. CLM-1822-H-0.1). Following activation, cells were washed with ice-cold 0.9% saline solution, and pellets were snap frozen before extraction in 1:1:1 pre-chilled methanol, HPLC-grade water (containing 1 μg/ml D6-glutaric acid) and chloroform. The extracts were shaken at 1400 rpm for 20 min at 4 °C and centrifuged at 16,000 g for 5 min at 4 °C. Then, 0.3 ml of the upper aqueous phase was collected and evaporated under vacuum. Metabolite derivatisation was performed using an Agilent autosampler. Dried samples were resuspended in 2% methoxamine in pyridine (40 μl, 1 h at 60 °C; Thermo Fisher Scientific, Cat# 25,104), followed by addition of N-(tert-butyldimethylsilyl)-N-methyl-trifluoroacetamide with 1% tert-butyldimethylchlorosilan (50 μl, 1 h at 60 °C). Derivatised samples were transferred to glass vials for analysis by gas chromatography mass spectrometry (GC–MS).

An Agilent 7890B GC (RRID:SCR_019449) equipped with a Rxi-5 ms column (Restek, UK) connected to a 5977A MSD (Agilent Technologies UK Limited, Stockport, UK) operating under electron impact ionization at 70 eV was used. The MS source was held at 230 °C, and the quadrupole at 150 °C. 1 μl of sample was injected in splitless mode with helium carrier gas at a rate of 1.0 ml/min. The initial GC oven temperature was held at 100 °C for 1 min before ramping to 160 °C at a rate of 10 °C/min, followed by a ramp to 200 °C at a rate of 5 °C/min and a final ramp to 320 °C at a rate of 10 °C/min with a 5 min hold. Compound detection was carried out in scan mode and analyte ion counts were normalised to the internal standard. A natural abundance correction was performed, and mass isotopomer distributions (MIDs) were calculated using in-house MATLAB scripts. Severely outlying data points were excluded from analysis; these are indicated in Supplementary Table S11.

### Glucose measurements

Supernatants were harvested from cells activated for 24 h and assayed for glucose concentration using a commercial blood glucose monitor (Sinocare, Safe-Accu 2). Four biological replicates in each of two independent experiments were performed.

### Lactate measurements

Supernatants were harvested from cells activated for 24 h and assayed for lactate concentration using a colorimetric lactate assay according to the manufacturer’s instructions (Sigma Aldrich, Cat # MAK064). Four biological replicates were included.

### Transcriptomic data analysis

#### Initial processing

Newly-synthesised, total and ribosome-protected mRNA-seq datasets were trimmed using Trim Galore v 0.5.0_dev (https://www.bioinformatics.babraham.ac.uk/projects/trim_galore/)^98^, and the quality validated using FastQC v0.11.8 (https://www.bioinformatics.babraham.ac.uk/projects/fastqc/)^99^. Reads were mapped to the GRCm38 mouse genome build using HISAT2 (v2.1.0)^100^, taking into account known splice sites for the GRCm38 Ensembl annotation release 90. For FPKM calculations, BAM files were first sorted and indexed using samtools v1.9^101^, and cuffnorm (Cufflinks v2.2.1)^102^ was then run with default parameters using the GRCm38 v90 annotation.

#### Differential expression analysis

Raw read counts were generated using Seqmonk v1.44.0 (https://www.bioinformatics.babraham.ac.uk/projects/seqmonk/)^103^ over mRNA (total, newly-synthesised mRNA) or CDS (ribosome-protected mRNA) for GRCm38 release 90. Read counts were filtered to include only those with a CDS (i.e., that are included in all three analyses). Genes located on the Y chromosome were also excluded due to the imbalance in sexes and mixed pools of mice used for some replicates. DESeq2 (v1.32.0) analysis^104^ for each of these was performed using default parameters, with ‘normal’ log2 fold change shrinkage. For newly-synthesised mRNA and ribosome profiling, samples were generated in two batches as indicated above, and this was included in the design formula; we noted a substantial batch effect for both datasets. Only genes with a base mean > 10 are visualised in plots and were used in downstream analyses. Z-scores for comparison across different data types were calculated as the shrunken log2 fold change normalised to the overall standard deviation for a given dataset. For the total mRNA sequencing, we noted that small differences in sample purity (90.9–92.3% CD4-positive cells in control samples, compared with 93.4–95% in dKO) resulted in lower detection of B cell-specific genes in the dKO (Supplementary Table S2); this complicates analyses of genes with decreased expression but has minimal impact on the increased genes on which we focussed our analysis.

#### Comparison of single and dKO mRNA abundance

Since the shrunken log2 fold changes calculated by DESeq2 are modelled based on the dataset being analysed, they are not directly comparable between models. For comparison of the mRNA abundance changes in single and double KO cells, we therefore used the raw log2 fold changes calculated by DESeq2. Z-scores were then calculated using the IntensityDiff R script (https://github.com/s-andrews/intensitydiff)^105^, which uses the local standard deviation of genes with a similar expression level for z-score calculation. The script was modified to allow the average expression and changes to be supplied directly, and to allow these to be provided for all three KOs together, such that the local standard deviations are modelled together, resulting in comparable z-scores. *P* values and FDR-adjusted p values were taken from the DESeq2 analysis.

#### mRNA stability estimation and identification of significant changes

mRNA stability for each gene was estimated essentially as described^106^, first using linear regression to estimate the correction factors for each sample based on the normalised read counts for newly-synthesised, pre-existing and total RNA fractions. The mean of the correction factors was then used to estimate mRNA half-lives for each gene. Since we noted a substantial batch effect between the two independent experiments in which the two replicate datasets were generated, the log2-fold change in half-life was calculated and normalised to the standard deviation for the two experiments separately before taking an average of these to give a standardised log2-fold change in stability.

Z-scores and p values for changes in stability between control and dKO were calculated using the IntensityDiff R script (https://github.com/s-andrews/intensitydiff)^105^, which uses the local standard deviation of genes with a similar expression level for z-score calculation. Since the stability estimates themselves do not reflect the expression level, the script was modified to allow direct input of expression levels (in this case, mean normalised read counts across total and newly-synthesised mRNA-seq libraries from which stability was estimated) and differences (change in stability in dKO vs control, i.e., the standardised log2-fold change in stability described above).

#### Hierarchical clustering and heatmap visualisation

Heatmaps were visualised in R using either heatmap.2 from the gplots package, or the pheatmap package. For plots in which the colour scale legend indicates that the fill represents log2 deviation from mean, the log2-fold difference between normalised read counts or metabolite abundance for each sample compared with the mean was calculated for each row separately. Otherwise, where z-scores are shown, these were calculated as described above, and no further scaling was performed. Where dendrograms are displayed, complete linkage clustering was performed using 1 minus the correlation coefficient as a distance metric; otherwise, no clustering was performed. For metabolic pathways, where multiple orthologues exist, genes exhibiting the highest expression are included. Annotations to the side indicate the mean total mRNA/metabolite abundance in control samples, summary quantitation of CLIP data as described below, and/or significantly altered metabolites, as indicated.

#### Visualisation of metabolic pathways

The z-score for the change in ribosome-protected mRNA for each gene was plotted as a dotplot using ggplot2, with diamond shaped points superimposed to represent the summary CLIP quantitation described below for the 72 h HITS-CLIP data. These were manually assembled into metabolic pathways in Illustrator.

#### Gene set analyses

GO term analysis was performed using GOrilla^107^ against the GO Biological Process terms, using default parameters, with all increased genes as defined for Fig. 2a (union of genes with log2-fold change > 0 and newly-synthesised mRNA FDR-adjusted *p* value < 0.1; total mRNA FDR-adjusted *p* value < 0.001; mRNA stability z-score > 2; or ribosome-protected mRNA FDR-adjusted *p* value < 0.05). The background list comprised all genes expressed with mean total mRNA abundance > 25 (normalised read counts) in CD4^+^ T cells. Terms with FDR-adjusted *p* value < 0.05 and enrichment > 2 were reduced by REVIGO^108^ based on semantic similarity, allowing ‘small’ similarity and prioritising terms with higher enrichment as representative terms.

The enrichment of transcription factor binding motifs amongst the genes in clusters 3, 4 and 5 (Fig. 2a) was assessed by gProfiler analysis^109^ against the TRANSFAC database, using the same background of expressed genes used for GOrilla analyses and default parameters. Multiple test correction was performed using the default g:SCS algorithm.

#### Designation of rate-limiting metabolic genes

Genes encoding enzymes and transporters involved in several metabolic pathways were designated as rate-limiting or non-rate-limiting based on literature searches, using a similar principle to that utilised for the RLEdb^110^, but performed manually and with higher stringency. Genes for which more than one publication could be found where it was referred to as rate-limiting or where a single publication demonstrated it to be so were designated as rate-limiting. If only a single publication referring to a gene as rate-limiting (but not demonstrating this) was found, the gene was not designated as such. In a few cases, publications were found referring to a given gene as non-rate-limiting; however, in most cases, genes are designated as non-rate-limiting due to an absence of evidence for a limiting role. The references supporting rate-limiting gene designation are shown in Supplementary Table S9.

### CLIP data analysis

#### Initial processing

For iCLIP datasets generated in this study, and published HITS-CLIP data for ZFP36 in CD4^+^ T cells activated for 4 h or 72 h + reactivation (downloaded from SRA; GSE96074)^46^, fastq files were uploaded to the Genialis iMaps platform (https://imaps.genialis.com/iclip)^111^, demultiplexed based on the first three bases, deduplicated based on their random 5' barcode (next four bases), and analysed using the iCount (v2.0.1.dev) demulitplex and analyse pipeline with default parameters to identify cross-link sites (https://icount.readthedocs.io/en/latest/index.html)^112,113^. For the iCLIP replicate where RNA from low- and high-molecular-weight protein:RNA complexes were sequenced separately, fastq files for these were merged prior to analysis. Significant crosslink sites were identified using the iCount peaks tool, both on individual replicate datasets and grouped replicates. For iCLIP data, peaks were also called on all replicates for both antibodies together.

#### Designation of targets

The gene and feature type for each crosslink site was assigned using the iCLIP_Genialis_feature_annotation R script available on GitHub/Zenodo (https://github.com/LouiseMatheson/Process_CLIP_data; https://doi.org/10.5281/zenodo.6525822)^114^, using the GRCm38 v90 annotation release to define transcripts/features and prioritising 3’UTR features.

For the 24 h iCLIP data, since the depth of coverage was relatively low, significant crosslink sites from grouped replicates were used. Genes were designated a target if a hit with FDR < 0.05 was detected with the grouped replicates for both antibodies for a given gene 3’UTR.

For HITS-CLIP data, significant crosslink sites from individual replicates were used. For each dataset, genes were designated a target if an identical significant crosslink site (FDR < 0.05) was found within its 3’UTR in at least two replicates (out of 5 or 4 for 4 h and 72 h data, respectively).

#### Summary quantitation and visualisation

To provide a summary quantitation for each dataset, the peak output for the merged replicates (with both antibodies together for iCLIP data) was used. The sum of reads over the 3’UTR at crosslink sites with an FDR < 0.25 was then calculated for each gene in a given CLIP dataset, providing a single value per gene to summarise the evidence for 3’UTR targeting of that gene. Where this is displayed in heatmaps, these sums were normalised to the 90th percentile of sums within a given dataset, considering only genes for which crosslinks were detected, to account for differences in depth of coverage in the different CLIP datasets, and log transformed. The colour scale for all CLIP annotations in all heatmaps is identical. Visualisation of the distribution of CLIP reads over selected genes included all reads from the merged dataset, without any filtering on FDR; plots were generated in R using ggplot2.

#### Integration with transcriptomic data

To assess whether CLIP targets were enriched within a given gene set (i.e., for the clusters identified in Fig. 2a, or (non-)rate-limiting genes), control gene sets were first generated that were matched in expression level and 3’UTR length to the gene set of interest, as follows: For each gene in the set, out of the remaining unassigned genes (i.e. not within the set of interest and not already chosen as a matched control for another gene in the set), we first selected genes with < 25% difference in 3’UTR length (or, if fewer than 1000, the 1000 with closest matched 3’UTR length). From these, we then selected the closest 100 in expression level (based on log2-transformed FPKM). From these, one gene was randomly selected as a matched control. Thus, the resulting control gene set will contain one matched control gene for each of the genes within the set of interest. This was done 100 times to generate 100 sets of control genes of equal size to the gene set of interest, and with very similar distribution in 3’UTR lengths and expression, albeit with overlap between the control sets. For each of the CLIP datasets, the RNA-seq libraries used for expression matching were from identical stimulation conditions: for HITS-CLIP, the corresponding RNA-seq datasets for 4 h and 72 h data were obtained from GSE96050^46^, and processed as described above for our mRNA-seq data to calculate FPKM.

The number of genes within each gene/control set that were CLIP targets or that contained a UAUUUAUU motif within their 3’UTR was then quantified, normalised to the median of the control sets and compared. The use of 100 control gene sets allowed us to calculate both the median and 5th/95th percentiles to estimate confidence intervals.

#### Sequence conservation of AREs

Orthologous sequences for 3’UTRs of interest were obtained using biomaRt^115^ or manually through the Ensembl web browser where this was not possible. Sequences encompassing the whole mouse 3’UTR and orthologous regions of several eutherian mammals (even if the 3’UTR annotation for a given species was shorter or absent) were aligned using Kalign^116^ with default parameters, before extracting the section of the alignment containing CLIP binding sites. In some alignments, the locations of gaps were manually adjusted to prioritise the alignment of AREs.

Conservation tracks displayed below visualisations of CLIP crosslinks over transcript 3’UTRs represent phyloP scores^117^ from a 60-way multiple alignment, obtained from the UCSC genome browser. Scores were averaged over a 7 bp sliding window.

### Quantification and statistical analysis

Statistical tests are indicated in the figure legends. Where data were approximately normally distributed, with similar variance, Student’s t-tests or ANOVA were used. For in vitro Th1 differentiation analyses, these assumptions were not met, and the nonparametric Kruskal–Wallis rank sum test was used. A *p* value < 0.05, with FDR correction for multiple testing where appropriate, was used to define a significant alteration unless indicated otherwise.

For transcriptomic analyses, DESeq2 was used for statistical analyses^104^, with the exception of mRNA stability, for which the IntensityDiff method was used (https://github.com/s-andrews/intensitydiff)^105^, as described in the relevant method details section. Since the different sensitivities of the transcriptomic methods resulted in very different numbers of genes passing the typical threshold of FDR < 0.05 and we wished to identify groups of genes with distinct regulatory patterns, we adjusted the significance threshold for each measurement to ensure that the genes with the most evidence for change in each of these were represented, even if some of these genes did not pass the threshold of 0.05. The thresholds chosen are detailed in the results section and indicated in Fig. 2a–d.

For gene set analyses, the statistical analyses implemented by GOrilla, REVIGO and gProfiler were used, with FDR (GOrilla/REVIGO) or g:SCS (gProfiler) correction.

For CLIP analysis of our own and published data, iCount was used to assign FDR-corrected *p* values to each crosslink site in each replicate or group of replicates. A gene bound over the 3’UTR was defined based on the consistency between replicates; see the relevant method details section.

Statistical enrichment of CLIP targets or UAUUUAUU motifs within gene sets of interest was assessed by comparison of the number of targets/genes with motifs with 100 control gene sets matched to the set of interest in expression and 3’UTR length, as detailed in the relevant method details section.

### Ethics approval and consent to participate

Procedures involving mice were approved by the Babraham Institute Animal Welfare, Experimentation and Ethics Committee and licenced under the Animals (Scientific procedures) Act 1986 and EU Directive 2010/63/EU.

## Supplementary Information

Supplementary Information 1.

Supplementary Information 2.

## Data Availability

All transcriptomic and iCLIP data generated as part of this study have been deposited in the GEO repository and are publicly available under the accessions GSE155087 and GSE214350 as of the date of publication (https://www.ncbi.nlm.nih.gov/geo/query/acc.cgi?acc=GSE155087; https://www.ncbi.nlm.nih.gov/geo/query/acc.cgi?acc=GSE214350). This paper also analyses existing, publicly available data, available on GEO under the accession GSE96076 (https://www.ncbi.nlm.nih.gov/geo/query/acc.cgi?acc=GSE96076). The metabolic profiling data reported in this paper are available in Supplementary Tables S10 and S11 of this paper; raw data will be shared by the corresponding authors upon reasonable request. All original code has been deposited on GitHub and released with Zenodo, and is publicly available as of the date of publication. Code for the overall analysis (ZFP36_L1_CD4_code v1.3) is available on Github (https://github.com/LouiseMatheson/ZFP36_L1_CD4_code) and released under the https://doi.org/10.5281/zenodo.7125361. Additionally, processing of CLIP data utilised code available on Github (https://github.com/LouiseMatheson/Process_CLIP_data) and released under the https://doi.org/10.5281/zenodo.6525822 (Process_CLIP_data v1.0). Any additional information required to reanalyse the data reported in this paper is available from the corresponding authors upon request.

## Abbreviations

3’UTR: 3’ Untranslated region
ARE: AU-rich element
dKO: *Zfp36/Zfp36l1* Double knockout
FCS: Fetal calf serum
GC–MS: Gas chromatography mass spectrometry
iCLIP: Individual nucleotide resolution crosslinking immunoprecipitation
LC–MS: Liquid chromatography mass spectrometry
PBS: Phosphate buffered saline
RBP: RNA-binding protein
TCA: Tricarboxylic acid
