# Supplementary material for: Multiomics analysis couples mRNA turnover and translational control of glutamine metabolism to the differentiation of the activated CD4+ T cell
Source: Sci Rep. 2022 Nov 16;12:19657. doi: 10.1038/s41598-022-24132-6 (PMC9669047; doi:10.1038/s41598-022-24132-6)
Supplement: Supplementary file 1 — Supplementary Information 1. [file 41598_2022_24132_MOESM1_ESM.pdf]

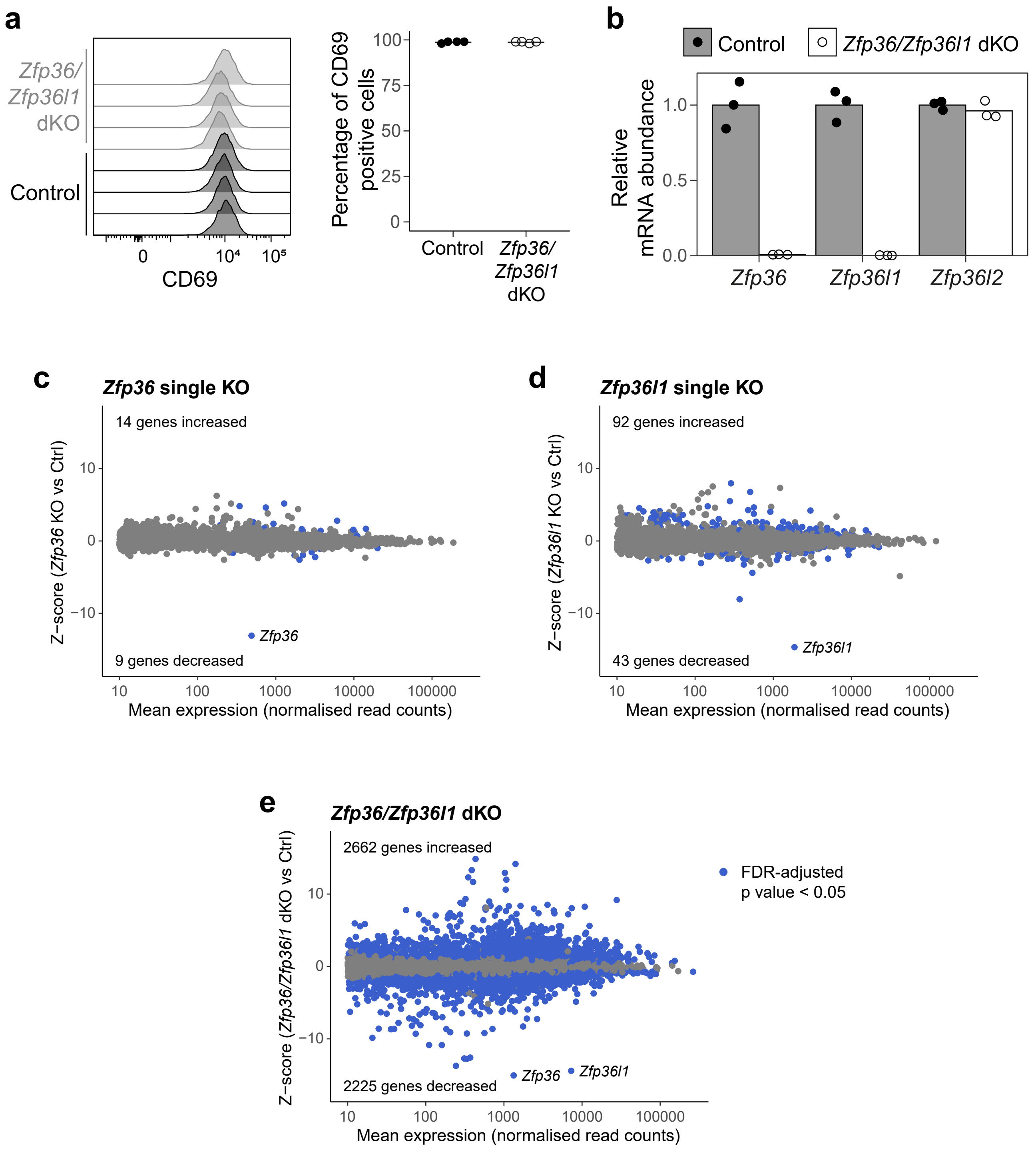

Figure S1. Efficient CD4-cre-mediated deletion of *Zfp36* and *Zfp36l1* does not affect cell activation (**a**) Flow cytometric quantitation of CD69 expression by control and *Zfp36/Zfp36l1* dKO $CD4^+$ T cells following 24h activation with anti-CD3 and anti-CD28. (**b**) Abundance of mRNA for Zfp36-family genes, measured by quantitative RT-PCR before and after activation of control and *Zfp36/Zfp36l1* dKO $CD4^+$ T cells with anti-CD3 and anti-CD28. For each gene, abundance is quantified relative to the mean abundance for control samples prior to activation. (**c-e**) Z-scores for the change in mRNA abundance in the indicated *Zfp36* and/or *Zfp36l1* KO $CD4^+$ T cells compared with corresponding floxed controls. Z-scores were calculated based on the standard deviation for genes with similar expression, considering all three models together; FDR-adjusted p values are from DESeq2 analysis.

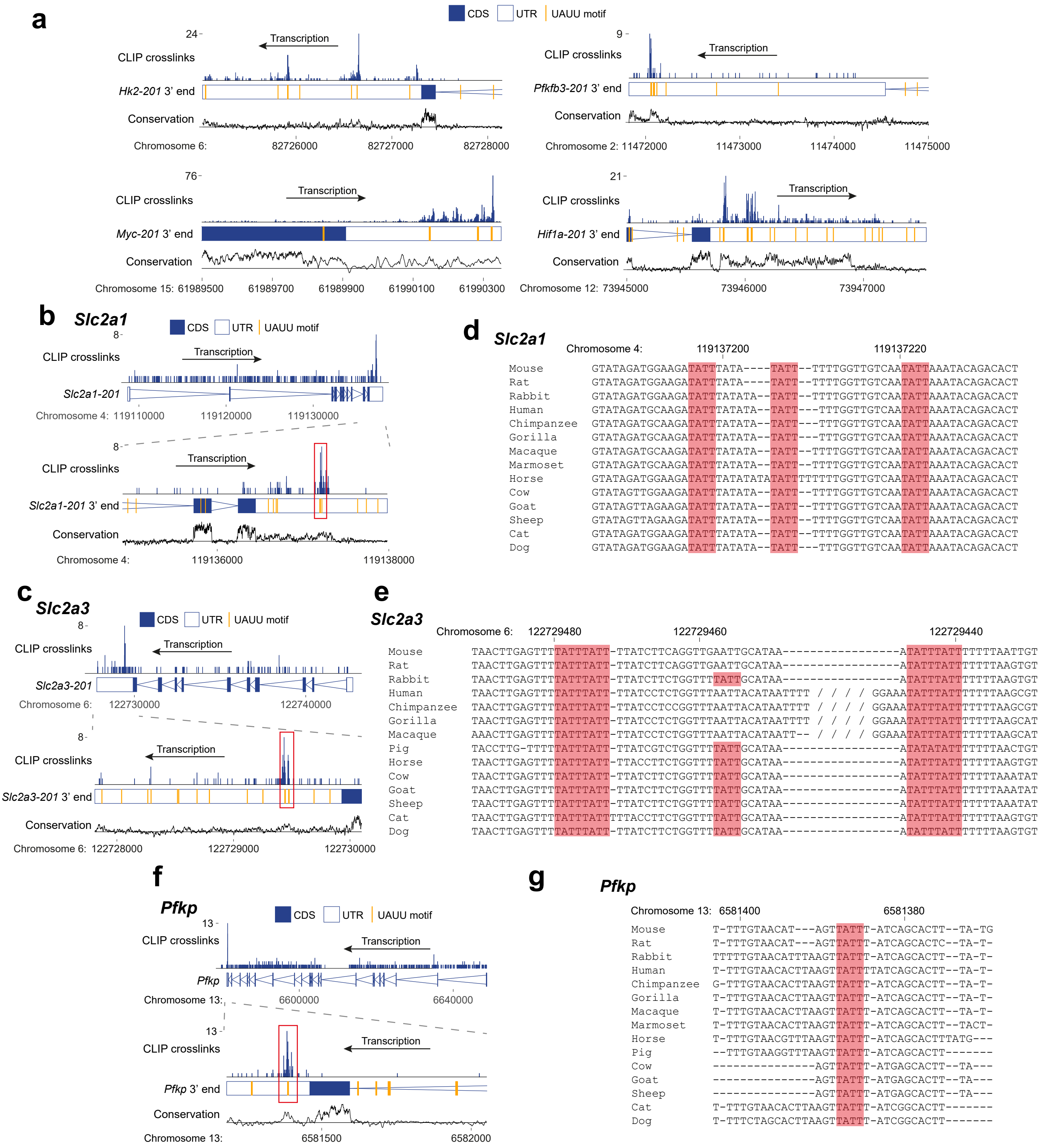

Figure S2. Direct targeting by ZFP36-family RBPs of genes encoding glycolytic enzymes.
(**a**) ZFP36/ZFP36L1 crosslinks in the 72h HITS-CLIP data over the 3'UTRs of the indicated transcripts. Occurrences of the UAUU motif are shown as vertical orange lines. Conservation tracks represent phyloP scores from a 60-way multiple alignment, averaged over a sliding 7 bp window. (**b-g**) ZFP36/ZFP36L1 crosslinks in the 72h HITS-CLIP data and multiple sequence alignments of the regions indicated by red boxes over the indicated *Slc2a1* (**b**, **d**), *Slc2a3* (**c**, **e**) and *Pfkp* (**f-g**) transcripts. For **b**, **c** and **f**, top panel depicts the whole transcript; bottom panel is zoomed in on the 3'UTR and depicted as described for **a**. In **d**, **e** and **g**, TATT (UAUU) motifs are highlighted, and genomic coordinates for the mouse 3'UTR are indicated.

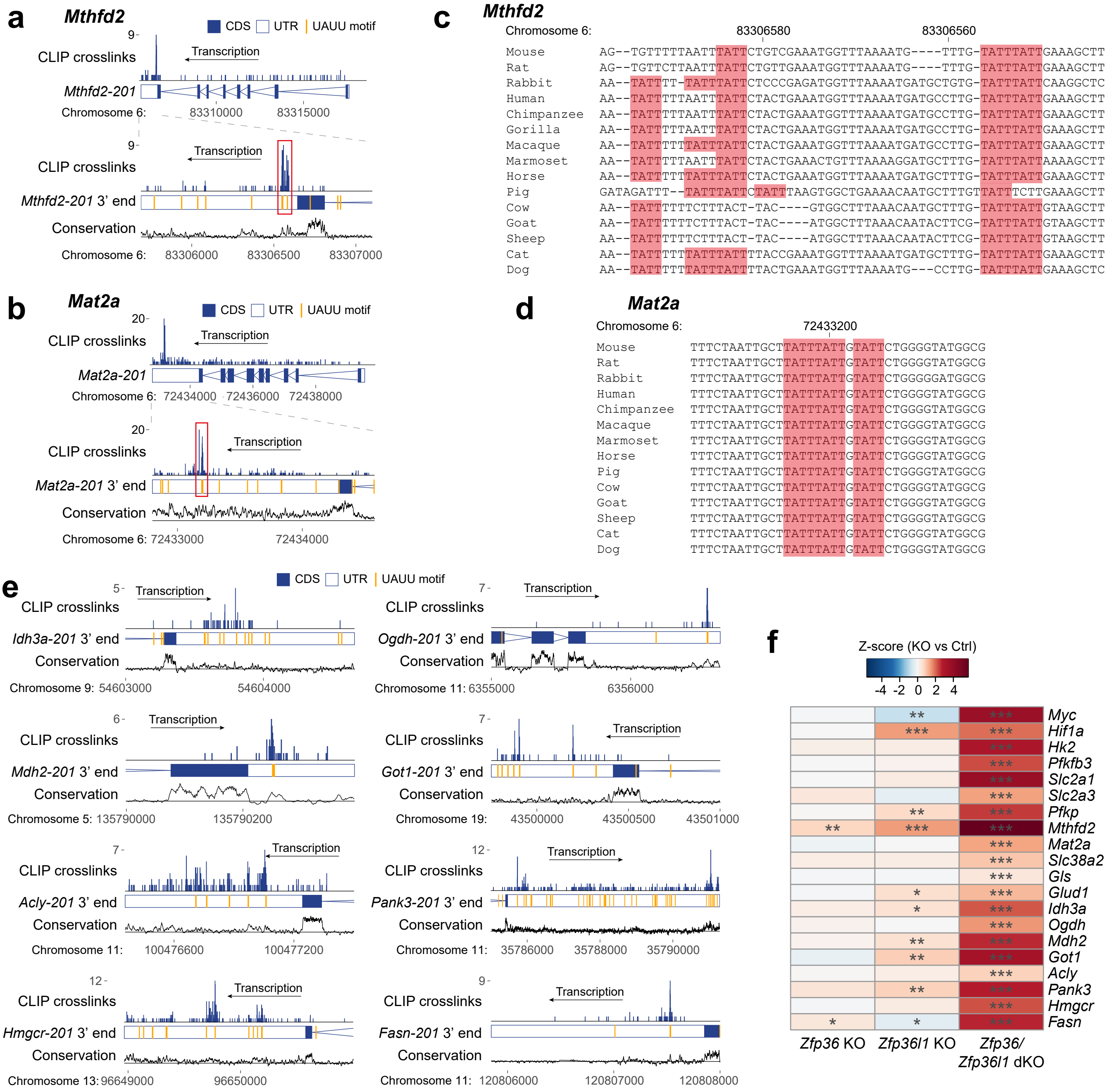

Figure S3. Regulation of metabolic genes by ZFP36-family RBPs (**a-d**) ZFP36/ZFP36L1 crosslinks in the 72h HITS-CLIP data and multiple sequence alignments of the regions indicated by red boxes over the indicated *Mthfd2* (**a**, **c**) and *Mat2a* (**b**, **d**) transcripts. For **a** and **b**, top panel depicts the whole transcript; bottom panel is zoomed in on the 3'UTR and additionally shows occurrences of the UAUU motif as vertical orange lines. Conservation tracks represent phyloP scores from a 60-way multiple alignment, averaged over a sliding 7 bp window. In **c** and **d**, TATT (UAUU) motifs are highlighted, and genomic coordinates for the mouse 3'UTR are indicated. (**e**) ZFP36/ZFP36L1 crosslinks in the 72h HITS-CLIP data over the 3'UTRs of the indicated transcripts, depicted as described for the bottom panel of **a** and **b**. (**f**) Heatmap showing z-scores for the change in mRNA abundance in the indicated *Zfp36* and/or *Zfp36l1* KO CD4^{+} T cells compared with corresponding floxed controls. Z-scores were calculated based on the standard deviation for genes with similar expression, considering all three models together. Significance from DESeq2 analysis is indicated: *** FDR-adjusted p value < 0.05; ** raw p value < 0.05; * raw p value < 0.1.

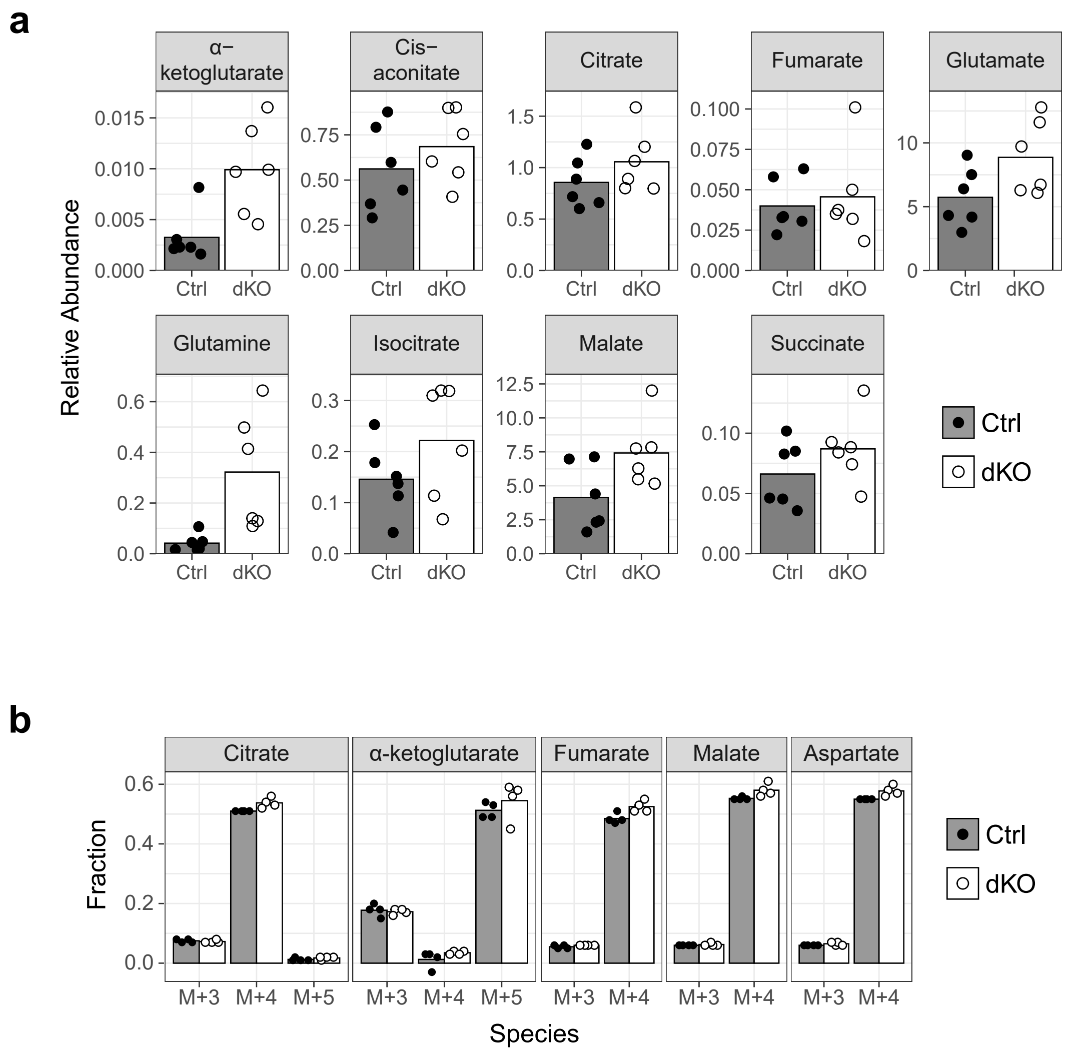

Figure S4. Perturbation of metabolites in ZFP36/ZFP36L1-deficient cells (**a**) Relative abundance of the indicated metabolites in dKO compared with control CD4^{+} T cells following 24 hours activation, meausred by LC-MS. (**b**) Fractional abundance of TCA cycle metabolites that had incorporated 3 (M+3 species), 4 (M+4) or 5 (M+5) labelled carbons following 24 hours activation of control and dKO CD4^{+} T cells in the presence of ^{13}C-labelled glutamine.

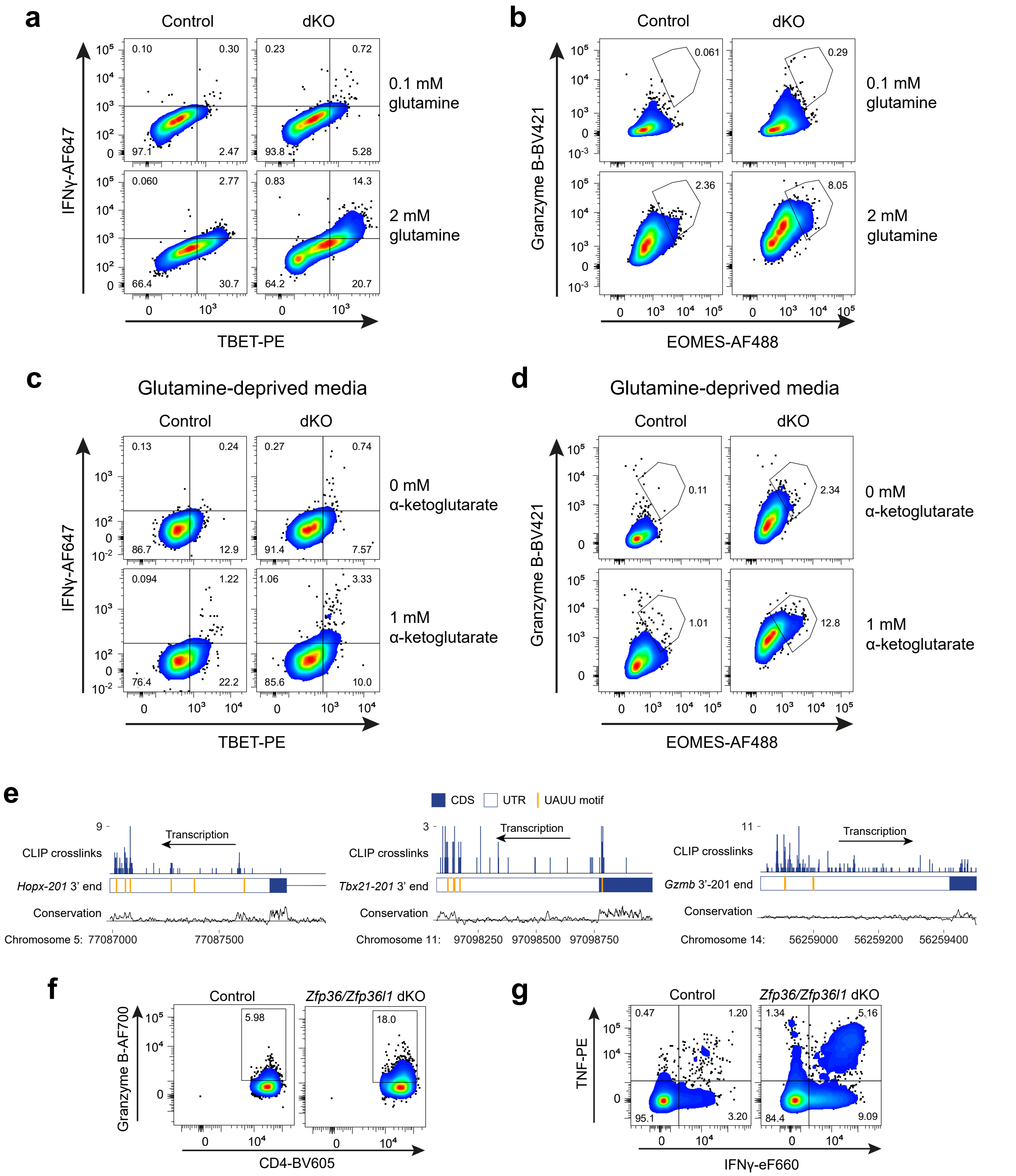

Figure S5. ZFP36 and ZFP36L1 impact CD4⁺ T cell differentiation. (**a-d**) Correspond to **Fig. 7a; b-c; d;** and **e**, respectively. Representative flow cytometry plots for one control and one dKO sample, showing TBET and IFNγ (**a**, **c**) or EOMES and Granzyme B (**b**, **d**) expression by CD4⁺ T cells following activation with anti-CD3 and anti-CD28 in Th1-polarising conditions for 24 hours, followed by a further 48 hours maintenance in IL-2, with varying concentrations of glutamine (**a-b**) or esterified α-ketoglutarate (**c-d**). In each case, representative plots for the maximum and minimum concentrations are shown. (**e**) ZFP36/ZFP36L1 crosslinks in the 72h HITS-CLIP data over the 3'UTRs of the indicated transcripts. Occurrences of the UAUU motif are shown as vertical orange lines. Conservation tracks represent phyloP scores from a 60-way multiple alignment, averaged over a sliding 7 bp window. (**f-g**) Correspond to **Fig. 7h-i** and **j-k**, respectively. Representative flow cytometry plots for one control and one dKO sample, showing EOMES and Granzyme B (**f**) or TNF and IFNγ (**g**) expression by CD4⁺ T cells isolated from the lung 10 days following infection with influenza A virus.
